# Knockin expression of human ADAMTS5 impairs cardiovascular development and aggravates cerebral cavernous malformations in mice

**DOI:** 10.1242/dmm.052668

**Published:** 2026-06-01

**Authors:** Xi Yang, Jieying Zhang, Zifeng Dai, Tianzi Yang, Liya Xie, Fei Gao, Xiangjian Zheng, Zhiming Han

**Affiliations:** ^1^ State Key Laboratory of Organ Regeneration and Reconstruction, Institute of Zoology, Chinese Academy of Sciences, Beijing 100101, China; ^2^ Department of Pharmacology and Tianjin Key Laboratory of Inflammation Biology, School of Basic Medical Sciences, and Center for Cardiovascular Diseases, Tianjin Medical University, Tianjin 300070, China; ^3^ Neurosurgery Department, The First Affiliated Hospital of Ningbo University, Ningbo 315010, China; ^4^ Beijing Institute for Stem Cell and Regenerative Medicine, Beijing 100101, China

**Keywords:** ADAMTS5, Versican, Cerebral cavernous malformation, Cardiac valve, Cardiac jelly, Endothelial cells

## Abstract

ADAMTS5 is an enzyme that cleaves chondroitin sulfate proteoglycans such as versican (VCAN) and aggrecan (ACAN). The *Adamts5^−/−^* mouse model exhibits aortic anomalies, and increased expression of *Adamts4* and *Adamts5* leads to excessive versican degradation and reduced cardiac jelly. In zebrafish, knockdown of *adamts5* rescues the cardiac phenotype conferred by *ccm1* deficiency in zebrafish embryos. Here, we generated an *ADAMTS5* knockin mouse model (*ADAMTS5^KI^*) to characterize the effect of induced expression of human ADAMTS5 on the cardiovascular system in mice. Sustained expression of ADAMTS5 in the endothelium diminished cardiac jelly formation and proteoglycan deposition in the atrioventricular cushion, and led to cardiac development arrest. Induced expression of ADAMTS5 in the endothelium of postnatal mice impaired cardiac valve patterning. Expression of ADAMTS5 in brain endothelial cells did not confer an obvious vascular defect. However, expression of ADAMTS5 in brain endothelial cells of *Ccm2*-deficient mice aggravated cerebral cavernous malformation (CCM) lesion burden and shortened the life span of *Ccm2*-deficient mice. These findings suggest that tight regulation of ADAMTS5 in the endothelium is essential for cardiovascular development and structural integrity, and that ADAMTS5 interacts with CCM signaling, contributing to CCM disease progression.

## Teaser

**Summary:** ADAMTS5 expression in the endothelium is essential for cardiovascular development and structural integrity, and ADAMTS5 interacts with cerebral cavernous malformation (CCM) signaling, contributing to CCM disease progression.

## INTRODUCTION

The extracellular matrix (ECM) constitutes a dynamic network that not only provides essential structural support for tissues but also orchestrates cellular behavior and participates in tissue repair and regeneration. Proteoglycans are critical ECM components and play an active role in maintaining structural and functional integrity (Zhao et al., 2025; Lockhart et al., 2011). The degradation of proteoglycans is tightly controlled by specific enzymes, the most well known among them being the ‘a disintegrin and metalloproteinase with thrombospondin motifs’ (ADAMTS) family (Stanton et al., 2011). ADAMTS5, a prominent member of the ADAMTS family, selectively cleaves chondroitin sulfate proteoglycans, such as versican (VCAN) and aggrecan (ACAN), exerting critical regulatory roles in cardiovascular development and disease progression (Kelwick et al., 2015; Barallobre-Barreiro et al., 2021).

Recent studies have indicated that ADAMTS5 is a crucial regulator of cardiovascular development and tissue homeostasis. In calcified human aortic valve tissues, the expression level of ADAMTS5 is markedly reduced (Li et al., 2017). In mice, genetic ablation of *Adamts5* leads to cardiac valve malformations, structural abnormalities in the aortic wall and heightened vascular calcification (Li et al., 2017; Zhu et al., 2025; Dupuis et al., 2019; Fava et al., 2018). Cardiac jelly, a critical structure of embryonic heart development, is essential for ventricular trabeculation, atrioventricular cushion morphogenesis and valve formation (Lockhart et al., 2011; Männer and Yelbuz, 2019). In the mouse heart, *Adamts5* expression was first detected in the myocardium under the outflow tract cushion at embryonic day (E) 11.5, and in the endocardium from E12.5 (Dupuis et al., 2011). This timing is consistent with the gradual disappearance of cardiac jelly from E11.5. Enhanced activities of ADAMTS4 and ADAMTS5 lead to excessive degradation of multifunctional proteoglycans, thereby disrupting normal cardiac jelly formation (Zhou et al., 2015). The specific role of ADAMTS5 expression in cardiovascular development, particularly in valvular morphogenesis, requires further investigation.

The cerebral cavernous malformation (CCM) pathway, mediated by the CCM1–CCM2–CCM3 protein complex, regulates vascular endothelial stability via downstream MEKK3–KLF2/4–ADAMTS signaling (Zhou et al., 2015, 2016; Hong et al., 2020). ADAMTS5, a key downstream effector of the CCM signaling pathway, plays a significant role in maintaining vascular homeostasis via its role in regulating proteoglycan degradation in the ECM environment (Fava et al., 2018; Dupuis et al., 2019; Barallobre-Barreiro et al., 2021). In zebrafish embryos, *adamts5* deletion mitigates cardiac abnormalities caused by *ccm1* deficiency (Zhou et al., 2015). In neonatal mice, endothelial-specific deletion of *Adamts5* reduces CCM lesion formation in white matter while increasing versican accumulation (Hong et al., 2020). Conversely, tetracycline-inducible *Adamts5* overexpression synergizes with *Ccm1* deficiency to exacerbate early CCM lesion formation (Hong et al., 2020). Reducing versican, an ADAMTS5 substrate, also reduces CCM lesion burden. This suggests that the cleavage product, versikine, could contribute to the severity of CCM lesion burden (Hong et al., 2020). However, the precise mechanistic role of ADAMTS5 and versican in CCM lesion progression across all CCM-deficient models has yet to be elucidated.

Here, we generated a human *ADAMTS5* knockin mouse model enabling conditional expression of ADAMTS5 in endothelial cells. The expression of ADAMTS5 in pan-endothelial cells or endocardial cells prevented cardiac jelly accumulation between the endocardium and the myocardium, led to proteoglycan disruption in endocardial cushions, arrested cardiac development, and ultimately led to embryonic lethality. ADAMTS5 overexpression restricted to brain endothelial cells did not cause overt vascular defects but significantly aggravated CCM lesions and reduced survival in *Ccm2*-deficient mice. These findings demonstrate that precisely regulated ADAMTS5 expression is essential for proper cardiovascular development, and ADAMTS5 functionally interacts with CCM signaling to promote CCM progression. This study provides new insights into the role of ADAMTS5 in cardiovascular biology and disease.

## RESULTS

### Sustained expression of *ADAMTS5* in endothelial cells results in the arrest of heart development associated with defective vascular circulation and reduced cardiac jelly

It has been reported that endocardial-specific loss of *Ccm1* increased expression of *Adamts4* and *Adamts5*, leading to premature degradation of cardiac jelly and embryonic lethality by the mid-gestation stage (Zhou et al., 2015). Deletion of *Adamts5*, but not *Adamts4*, alleviated CCM lesion burden in the *Ccm2*-deficient CCM mouse model (Hong et al., 2020). To further investigate the role of ADAMTS5 in the endothelium, we generated *ADAMTS5* knockin mice by inserting a floxed cassette of stop sequences followed by sequences encoding human *ADAMTS5* and *EGFP* in the *Rosa26* gene locus, hereafter termed *ADAMTS5^KI^* (Fig. S1A). To test the effectiveness of this knockin allele in the endothelial lineage, we crossed *ADAMTS5^KI^* mice with *Tie2-Cre* mice to produce *Tie2-Cre;ADAMTS5^KI/+^* mice. Quantitative PCR (qPCR) analysis of the whole embryo confirmed a significant elevation of *ADAMTS5* expression in *Tie2-Cre;ADAMTS5^KI/+^* mice compared with that in littermate controls (Fig. S1B). GFP staining demonstrated that EGFP is expressed in the endothelium, including the developing vessels and endocardium (Fig. S1C).

**Fig. 1.**
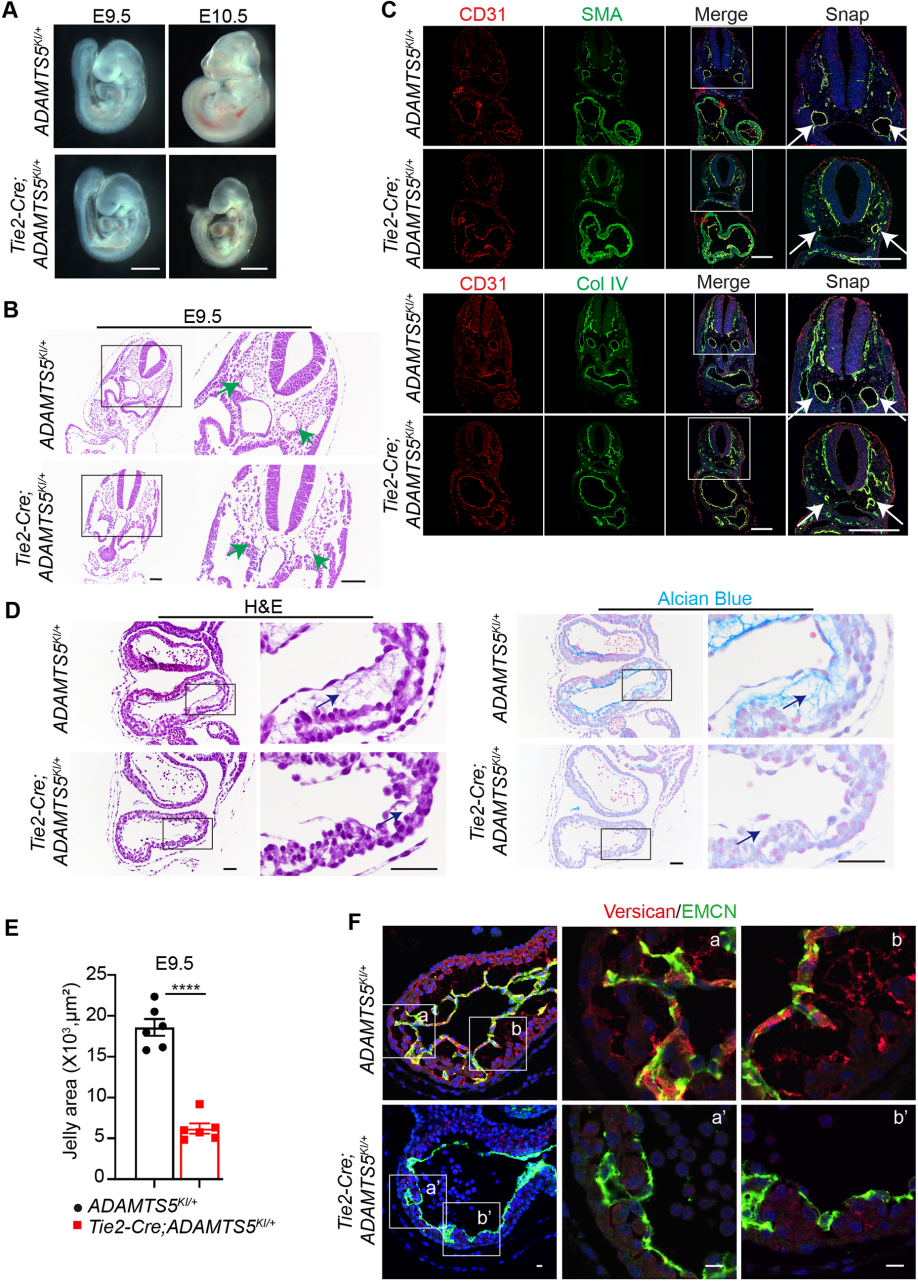
**Increased expression of ADAMTS5 in the endothelium results in vascular and heart defects during embryonic development.** (A) Representative stereomicroscopic images of *Tie2-Cre;ADAMTS5^KI/+^* embryos and littermate controls at embryonic day (E) 9.5 and E10.5. Scale bars: 1 mm. (B) Hematoxylin and Eosin (H&E) staining of transverse sections of E9.5 *ADAMTS5^KI/+^* and *Tie2-Cre;ADAMTS5^KI/+^* embryos shows the presence of normally lumenized dorsal aortas in the *ADAMTS5^KI/+^* embryos but not in *Tie2-Cre;ADAMTS5^KI/+^* embryos. Green arrows indicate dorsal aortas. Scale bars: 50 μm. (C) Co-immunostaining of CD31 with smooth muscle actin (SMA, top) and collagen IV (Col IV, bottom) on transverse sections of E9.5 *ADAMTS5^KI/+^* and *Tie2-Cre;ADAMTS5^KI/+^* embryos. Panels on the right show higher-magnification views. White arrows indicate the dorsal aortas. Scale bars: 100 μm. (D) H&E and Alcian Blue staining display reduced cardiac jelly in *Tie2-Cre;ADAMTS5^KI/+^* embryos. Panels on the right show higher-magnification views. Blue arrows indicate the reduced cardiac jelly between endocardium and the myocardium. Scale bars: 50 μm. (E) Quantification of the cardiac jelly area of E9.5 hearts. *n*=6, six sections analyzed for each group. Data are presented as mean±s.e.m., and statistical significance was determined using an unpaired two-tailed Student's *t*-test. ****P<0.0001*.* (F) Co-immunostaining for endomucin (EMCN) and versican on transverse sections of E9.5 *ADAMTS5^KI/+^* and *Tie2-Cre;ADAMTS5^KI/+^* embryos. Panels on the right show higher-magnification views. Scale bars: 10 μm. In C,F, nuclear staining is shown in blue. Images are representative of *n*=5-6 for each group.

To assess the effects of continuous endothelial *ADAMTS5* expression on cardiovascular development, we observed the morphology and quantified the number of embryos. No live *Tie2-Cre;ADAMTS5^KI/+^* mice were detected among 48 postnatal offspring from this cross (Table 1). Timed mating revealed that live *Tie2-Cre;ADAMTS5^KI/+^* embryos displayed modest developmental delay (∼2 somites) but comparable gross morphology to littermate controls at E9.5. By E10.5, all *Tie2-Cre;ADAMTS5^KI/+^* embryos were dead (Table 1, Fig. 1A). Thus, continuously increased endothelial expression of *ADAMTS5* results in embryonic lethality during early gestation.

**Table 1.** Offspring of *Tie2-Cre* and *ADAMTS5^KI/+^* mating at various developmental stages

Genotype	Age				
	E9.5	E10.5	E12.5	E15.5	P1-P21
Wild type	28	22	7	9	13
*Tie2*-*Cre*	26	13	4	6	17
*ADAMTS5^KI/+^*	17	18	12	6	18
***Tie2-Cre;ADAMTS5^KI/+^***	**16 (4 dead)**	**17 (17 dead)^a^**	**6 (6 dead)^a^**	**0^a^**	**0^a^**
Total	87	70	29	21	48

Viability of *Tie2*-*Cre;ADAMTS5^KI/+^* mice at different developmental stages is given in parentheses. Bold values indicate the numbers of mutant embryos. ^a^*P*<0.001, χ^2^ test.

Histological analysis of E9.5 *Tie2-Cre;ADAMTS5^KI/+^* and littermate control embryos revealed that *Tie2-Cre;ADAMTS5^KI/+^* embryos failed to form a patent dorsal aorta (Fig. 1B). Immunostaining for CD31 (also known as PECAM1), smooth muscle actin (SMA, also known as ACTA2) and collagen IV demonstrated that endothelial cells, vascular smooth muscle cells and basement membrane components were appropriately differentiated and spatially localized; however, these analyses confirmed a markedly constricted dorsal aorta lumen in *Tie2-Cre; ADAMTS5^KI/+^*embryos (Fig. 1C). Impaired lumenization of dorsal aorta likely prevented the establishment of a functional circulation system. In contrast, the vessel patterning of the yolk sac appeared grossly normal (Fig. S1D).

Hematoxylin and Eosin (H&E) staining of the developing heart suggested a reduction in cardiac jelly in the space between the endocardium and the myocardium in hearts of *Tie2-Cre; ADAMTS5^KI/+^*embryos (Fig. 1D). Alcian Blue staining revealed the loss of cardiac jelly wrapping around the trabeculae in the hearts of *Tie2-Cre;ADAMTS5^KI/+^*embryos (Fig. 1D). Quantitative analyses demonstrated a significant decrease in cardiac jelly area in *Tie2-Cre;ADAMTS5^KI/+^* embryos compared with that in littermate controls (Fig. 1E). Immunostaining for endomucin (EMCN), an endothelial marker, and versican, a major proteoglycan component of cardiac jelly, showed that versican was nearly undetectable in *Tie2-Cre;ADAMTS5^KI/+^* hearts (Fig. 1F). Thus, endothelial expression of ADAMTS5 results in early-gestational heart failure associated with impaired blood circulation and loss of cardiac jelly integrity.

### Sustained expression of *ADAMTS5* in endocardial cells results in cardiac development arrest associated with reduced cardiac jelly

ADAMTS5 cleaves the proteoglycan versican, which is a crucial component of cardiac jelly (Kelwick et al., 2015). To determine whether the increased expression of ADAMTS5 in endocardial cells would be sufficient to induce cardiac jelly degradation and affect heart development, we crossed *ADAMTS5^KI^* mice with *Nfatc1-Cre* mice to express ADAMTS5 in the endocardial lineage. qPCR analysis of E11.5 hearts showed a significant increase in ADAMTS5 expression (Fig. S2A). Immunostaining for GFP showed the specific knockin expression of ADAMTS5 in the endocardium (Fig. S2B). Timed mating demonstrated that there were no differences in gross morphology between live *Nfact1-Cre;ADAMTS5^KI/+^* embryos and littermate control embryos before E11.5, even though *Nfatc1-Cre;ADAMTS5^KI/+^* embryos started dying at E10.5, and all embryos were dead by E12.5 (Table 2). Thus, endocardial expression of ADAMTS5 results in embryonic lethality during mid-gestation.

**Table 2.** Offspring of *Nfatc10Cre* and *ADAMTS5^KI/+^* mating at various developmental stages

Genotype	Age					
	E9.5	E10.5	E11.5	E12.5	E15.5	P1-P21
Wild type	13	26	16	5	20	15
*Nfatc1-Cre*	10	21	11	7	10	15
*ADAMTS5^KI/+^*	10	16	6	7	15	10
***Nfatc1-Cre;ADAMTS5^KI/+^***	**10**	**8 (2 dead)**	**7 (3 dead)**	**5 (5 dead)^a^**	**0^a^**	**0^a^**
Total	43	71	40	24	45	40

Viability of *Nfatc1*-*Cre;ADAMTS5^KI/+^* mice at different developmental stages stages is given in parentheses. Bold values indicate the numbers of mutant embryos. ^a^*P*<0.001, χ^2^ test.

**Fig. 2.**
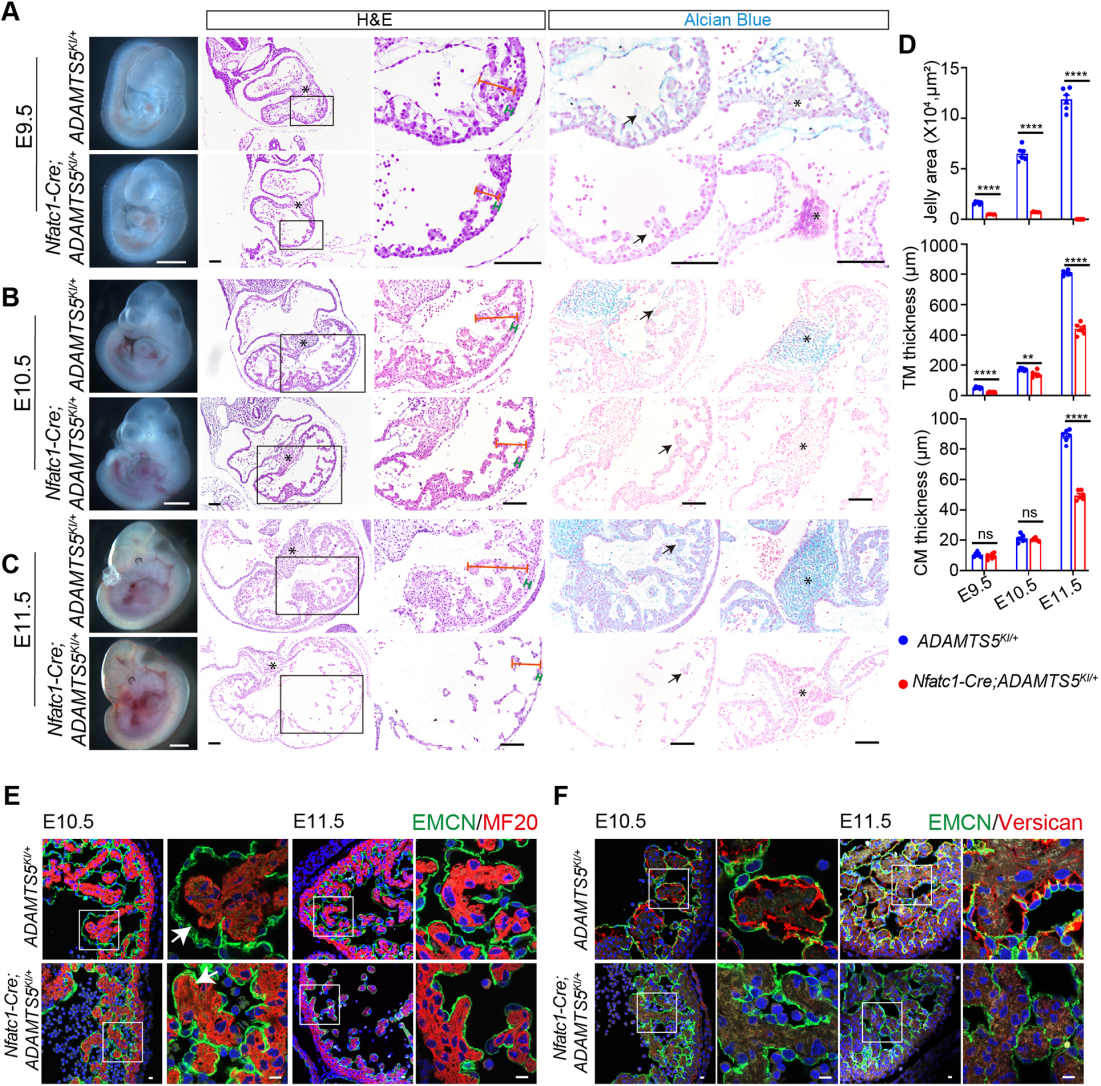
**Endocardial expression of *ADAMTS5* impairs heart development.** (A-C) Representative stereomicroscopic images, H&E and Alcian Blue staining of embryos and cross-sections of embryos at E9.5 (A), E10.5 (B) and E11.5 (C) showing defective heart development of *Nfact1-Cre;ADAMTS5^KI/+^* mice as gestation progresses. H&E staining shows a thin trabeculae myocardium (TM, orange bars) and compacted myocardium layer (CM, green bars) of *Nfact1-Cre;ADAMTS5^KI/+^* hearts. Alcian Blue staining displays reduced cardiac jelly at the TM (indicated by black arrows) and atrioventricular cushion (indicated by asterisks) in *Nfact1-Cre;ADAMTS5^KI/+^* mice. Panels on the right show higher-magnification views. Scale bars: 100 μm. (D) Quantitation plots of jelly area, and TM and CM thickness from H&E- and Alcian Blue-stained sections (E9.5, *n*=6; E10.5, *n*=6; E11.5, *n*=6). Data are presented as mean±s.e.m., and statistical significance was determined using an unpaired two-tailed Student's *t*-test. ns, not significant; **P<0.01; ****P<0.0001. (E,F) Co-immunostaining of endomucin (EMCN) and MF20 (E) shows a reduced endocardial-myocardial space in E10.5 and E11.5 *Nfact1-Cre;ADAMTS5^KI/+^* hearts. White arrows in E indicate the endocardial-myocardial space. Co-immunostaining of EMCN and versican (F) shows the reduction of versican in the endocardial-myocardial space in E10.5 and E11.5 *Nfact1-Cre;ADAMTS5^KI/+^* hearts. Nuclear staining is shown in blue. Panels on the right show higher-magnification views. Scale bars: 10 μm. Images are representative of *n*=5-6 for each group.

H&E staining revealed diminished cardiac jelly, fewer myocardial trabeculae and thinner myocardium in *Nfatc1-Cre;ADAMTS5^KI/+^* hearts at E9.5, in comparison to well-formed trabeculae and compacted myocardium in littermate controls (Fig. 2A). These phenotypes become more pronounced in *Nfatc1-Cre;ADAMTS5^KI/+^* hearts at E10.5 and E11.5 (Fig. 2B,C). Alcian Blue staining showed a lack of cardiac jelly in the hearts of *Nfatc1-Cre;ADAMTS5^KI/+^* embryos at each time point. The space between the myocardium and endocardium was filled with proteoglycan-cardiac jelly in control hearts, but this was missing in *Nfatc1-Cre;ADAMTS5^KI/+^* hearts. We also observed ventricular chamber dilation in the histology results for *Nfatc1-Cre;ADAMTS5^KI/+^* embryos at E11.5, but the phenotype was not marked before E11.5 (Fig. 2A-C).

Previous studies indicate that *Adamts5* is expressed during cardiac valve maturation in regions where versican cleavage fragments are detected (Cikach et al., 2018). *Adamts5-*null embryos had enlarged and malformed pulmonary valves, and adult *Adamts5^−/−^* mice have myxomatous pulmonary, aortic and mitral valves, which present significant thickening of pulmonary valve cusps, and aortic and mitral valves (Dupuis et al., 2011). Consistent with these observations, Alcian Blue staining also confirmed the loss of jelly glycosaminoglycans surrounding the atrioventricular cushion in the *Nfatc1-Cre;ADAMTS5^KI/+^* hearts (Fig. 2A-C; Fig. S2C). Quantitation of the cardiac jelly area around the trabeculae of E9.5 hearts revealed >70% reduction, and analyses of E10.5 and E11.5 hearts showed even greater reductions (>90% and >95%, respectively) in *Nfatc1-Cre;ADAMTS5^KI/+^* mice compared to littermate controls (Fig. 2D). Reduction of trabeculae and myocardium thickness demonstrated impaired heart development in *Nfatc1-Cre;ADAMTS5^KI/+^* mice, despite the myocardium thickness still increasing during embryonic development (Fig. 2D). Co-immunostaining for endomucin and myocardium marker MF20 antibody also showed the reduction of the endocardial-myocardial space, and endomucin and versican co-immunostaining showed the lacking of versican in *Nfatc1-Cre;ADAMTS5^KI/+^* hearts (Fig. 2E,F). Thus, sustained endocardial expression of ADAMTS5 results in mid-gestation heart failure associated with reduced cardiac jelly and impaired myocardium growth.

### Induced expression of ADAMTS5 in the endothelium of postnatal mice results in abnormal cardiac valve maturation

**Fig. 3.**
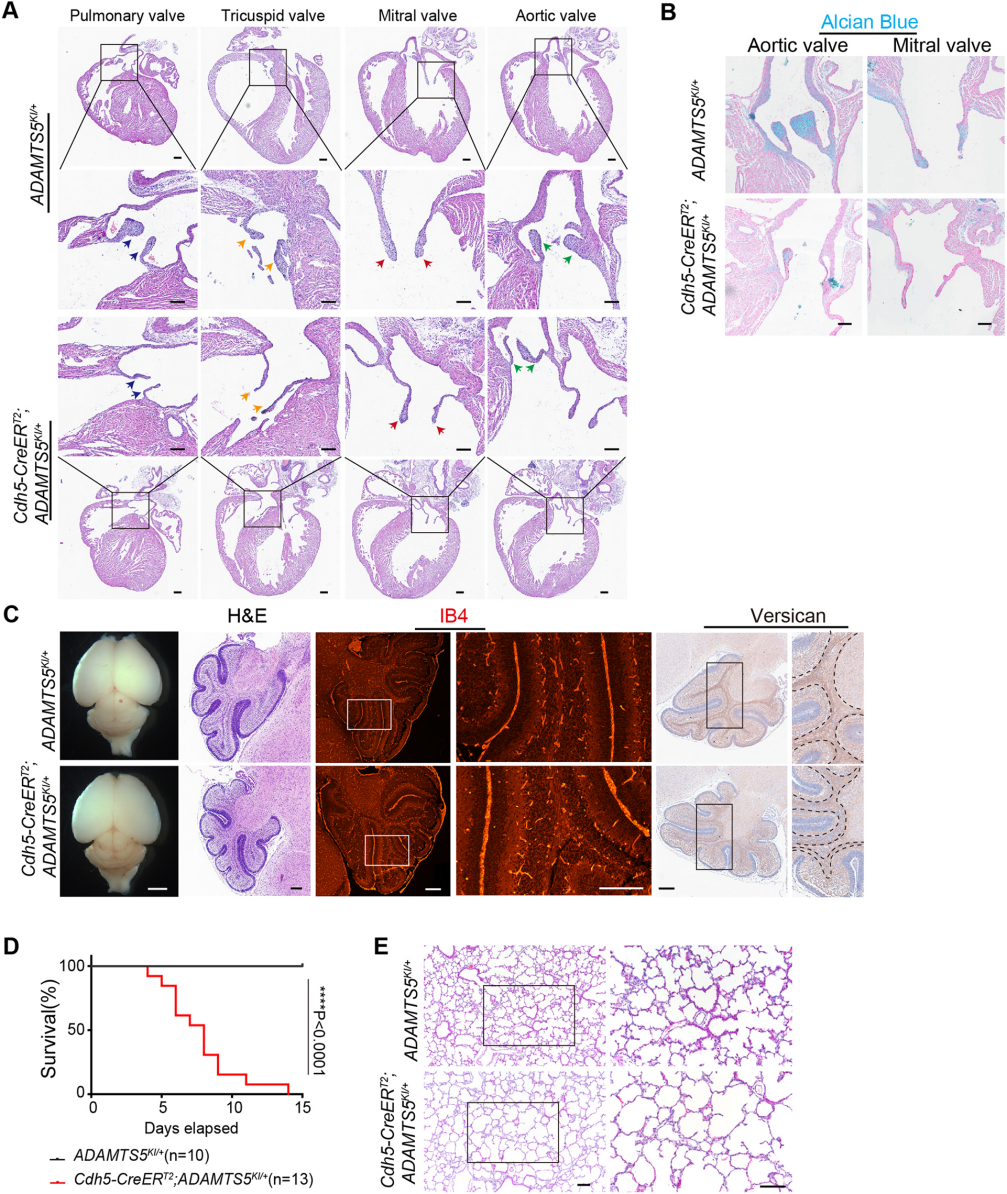
**Induced expression of ADAMTS5 in endothelial cells impairs cardiac valve development.** (A) Pups were intragastrically injected with 4-hydroxytamoxifen at postnatal day (P) 1, and the heart tissues were harvested at P8. H&E staining of heart tissues reveals significant thinning of pulmonary valves (blue arrows), tricuspid valves (orange arrows), mitral valves (red arrows) and aortic valves (green arrows) in *Cdh5-CreER^T2^;ADAMTS5^KI/+^* mice compared with those in *ADAMTS5^KI/+^* mice. Scale bars: 200 μm. Middle panels show higher-magnification views. Scale bars: 100 μm. (B) Alcian Blue staining indicates the reduced extracellular matrix (proteoglycan) in aortic and mitral valves of *Cdh5-CreER^T2^;ADAMTS5^KI/+^* hearts. Scale bars: 200 μm. (C) Representative micrographs, H&E staining and immunostaining of brain sections at P8. Stereomicroscopic images and H&E staining display no bleed or cerebral cavernous malformation (CCM) lesion in *Cdh5-CreER^T2^;ADAMTS5^KI/+^* mice. Isolectin B4 (IB4) staining shows no significant change in vascular density of induced *Cdh5-CreER^T2^;ADAMTS5^KI/+^* mice. Immunostaining of versican is reduced in induced *Cdh5-CreER^T2^;ADAMTS5^KI/+^* mice brains compared with that in *ADAMTS5^KI/+^* control mice brains. Dotted lines mark white matter. Panels on the right show higher-magnification views. Scale bars: 2 mm (micrographs); 200 μm (H&E and immunostained images). (D) Postnatal survival curve of induced *Cdh5-CreER^T2^;ADAMTS5^KI/+^* (*n*=13) and *ADAMTS5^KI/+^*(*n*=10) mice. Statistical analysis was performed using the Mantel–Cox test. ****P<0.0001. (E) H&E staining of lung tissues showed reduced alveolar number in induced *Cdh5-CreER^T2^;ADAMTS5^KI/+^* mice compared with *ADAMTS5^KI/+^* mice. Panels on the right show higher-magnification views. Representative images from at least three or more independent experiments are shown. Scale bars: 100 μm.

Due to the lethality of embryos with sustained ADAMTS5 expression in the endothelium, we crossed *ADAMTS5^KI^* mice with *Cdh5-CreER^T2^* mice to generate *Cdh5-CreER^T2^;ADAMTS5^KI/+^* mice, enabling postnatal induction of ADAMTS5 expression. qPCR and western blot analysis confirmed a significant increase of ADAMTS5 and GFP expression in 4-hydroxytamoxifen-treated *Cdh5-CreER^T2^;ADAMTS5^KI/+^* mice compared with that in littermate controls (Fig. S3A,B). GFP immunostaining of heart and brain sections demonstrated efficient endothelial-specific recombination of the *ADAMTS5* knockin allele following induction (Fig. S3C,D). Consistent with the reduced proteoglycan phenotype observed in the atrioventricular cushion of *Nfact1-Cre;ADAMTS5^KI/+^* embryos (Fig. 2A-C), postnatal endothelial induction of ADAMTS5 expression resulted in marked thinning of the pulmonary, aortic, mitral and tricuspid valves (Fig. 3A). Alcian Blue staining revealed decreased proteoglycan content in the aortic and mitral valves (Fig. 3B), suggesting that excessive ADAMTS5 activity leads to proteoglycan depletion and consequent structural attenuation of cardiac valves.

Although *Adamts5* has been implicated as a downstream effector of CCM signaling, whether it is causal for CCM has not been clearly established. Gross images, H&E staining and isolectin B4 staining of brains revealed no evidence of hemorrhage or CCM lesions in either the cerebrum or cerebellum of induced *Cdh5-CreER^T2^;ADAMTS5^KI/+^* mice (Fig. 3C). Immunostaining demonstrated reduced versican levels in 4-hydroxytamoxifen-treated *Cdh5-CreER^T2^; ADAMTS5^KI/+^* mice compared to *ADAMTS5^KI/+^* controls (Fig. 3C).

Survival curves showed that the induction of ADAMTS5 from postnatal day (P) 1 caused premature death of *Cdh5-CreER^T2^;ADAMTS5^KI/+^* mice within 2 weeks after birth (Fig. 3D). Major organs were harvested at P8 to assess the potential cause of lethality. Histological analysis demonstrated marked alveolar dilation in the lungs of induced *Cdh5-CreER^T2^;ADAMTS5^KI/+^* mice (Fig. 3E). Immunostaining further revealed a reduction of type II alveolar epithelial cells and proliferative cells compared with those in littermate controls (Fig. S4A), suggesting impaired pulmonary development or maintenance. The gross images and H&E staining of *Cdh5-CreER^T2^;ADAMTS5^KI/+^* mice showed no histological defect in the kidney, but there were bleeding lesions and increased inflammatory cell infiltration in the spleen and liver, respectively (Fig. S4B-D).

### Induced expression of ADAMTS5 in brain endothelial cells does not cause vascular abnormality

**Fig. 4.**
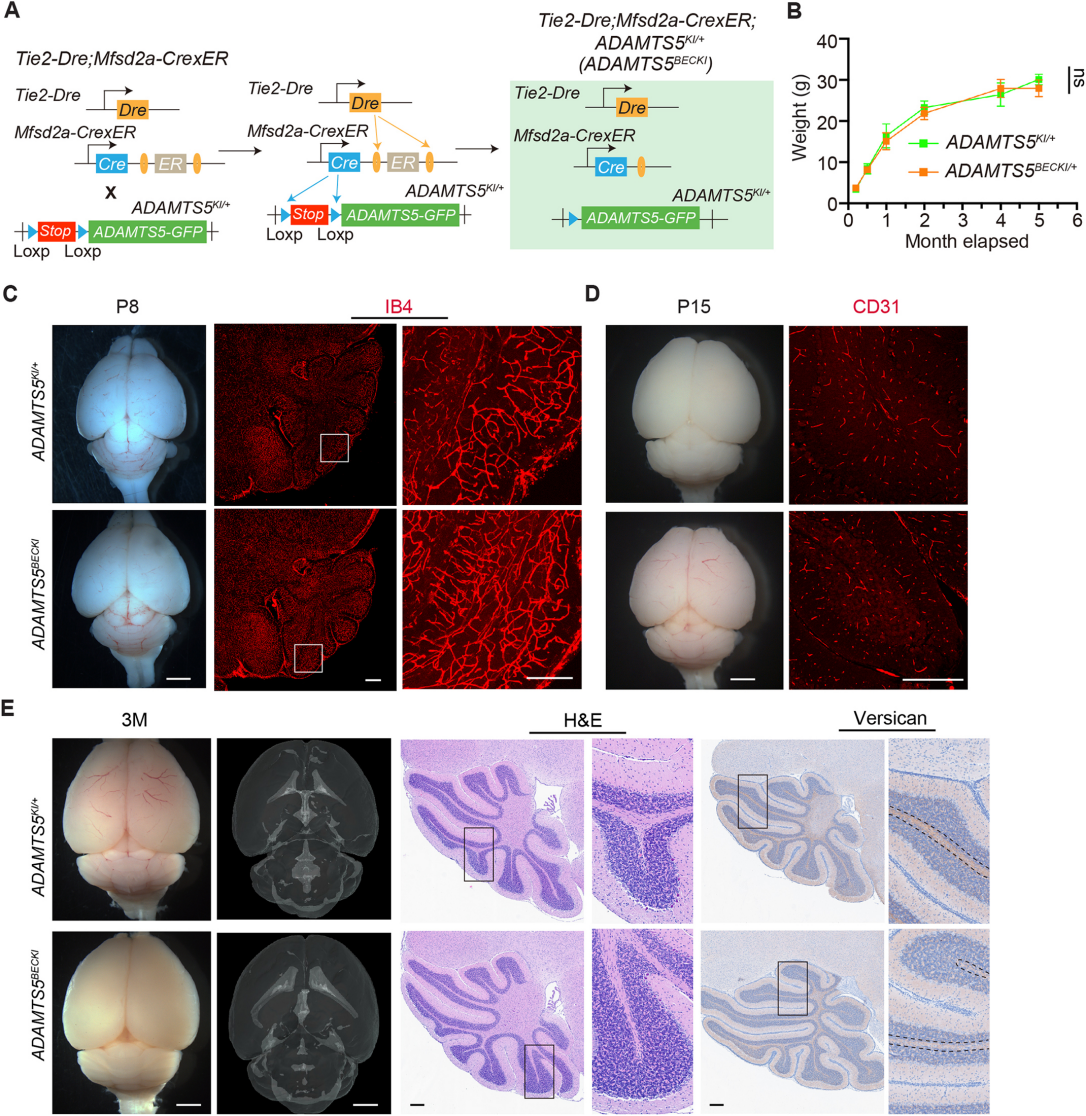
**Knockin expression of ADAMTS5 in brain endothelial cells does not cause vascular abnormality.** (A) Schematic of the genetic cross to generate *ADAMTS5^BECKI^* mice with sustained ADAMTS5 expression in brain endothelial cells. (B) Body weight gain of *ADAMTS5^BECKI^* mice and littermate controls (4-6 mice/group). Data are presented as mean±s.e.m. and statistical significance was determined using an unpaired two-tailed Student's *t*-test. ns, not significant. (C) Representative micrographs revealed normal vessel development in *ADAMTS5^BECKI^* and *ADAMTS5^KI/+^* control mice at P8. Isolectin B4 (IB4) staining of vibratome sections indicates normal vessel density of *ADAMTS5^BECKI^* mice compared with that of littermate controls. (D) Representative micrographs and CD31 immunostaining showing no CCM lesion and normal vessel density in an *ADAMTS5^BECKI^* brain at P15. (E) Representative brain micrographs, micro-CT images and H&E staining of brain sections of 3-month-old *ADAMTS5^BECKI^* and *ADAMTS5^KI/+^* mice showed no malformed vascular lesions. Versican immunostaining was reduced in *ADAMTS5^BECKI^* mice compared with that in littermate controls. Dotted lines mark white matter. Panels on the right show higher-magnification views. Representative images from at least three or more independent experiments are shown. Micrographs and CT Scale bars: 2 mm (micrographs and micro-CT images); 200 μm (H&E and immunostained images).

*Adamts5* is a downstream target of CCM signaling (Zhou et al., 2016; Hong et al., 2020), but it remains unclear whether increased expression of ADAMTS5 is sufficient to cause brain vascular malformation. We used the *Tie2-Dre* and *Mfsd2a-CrexER* (Dre-Cre) duo recombination system to drive ADAMTS5 expression specifically in endothelial cells of the central nervous system without the requirement of tamoxifen induction (Yang et al., 2022). We generated *Tie2-Dre;Mfsd2a-CrexER;ADAMTS5^KI/+^* (thereafter *ADAMTS5^BECKI^*, where BEC refers to brain endothelial cells) mice (Fig. 4A). The dual-recombinase system effectively drove the recombination of the *ADAMTS5* knockin allele in brain endothelial cells as indicated by GFP stainings (Fig. S5). *ADAMTS5^BECKI^* mice appeared grossly normal and with normal body weight gain up to 5 months of age (Fig. 4B). Micrographs of P8 and P15 *ADAMTS5^BECKI^* brains and micro-computed tomography (CT) imaging of 3-month-old *ADAMTS5^BECKI^* brains did not detect any bleeding or malformed vascular lesion in the cerebrum and cerebellum, consistent with the isolectin B4, CD31 and H&E staining results (Fig. 4C-E). Versican immunostaining was reduced in *ADAMTS5^BECKI^* mice compared to that in *ADAMTS5^KI/+^* mice (Fig. 4E). These results indicate that increased expression of ADAMTS5 in brain endothelial cells does not impair normal vessel development.

### Expression of ADAMTS5 synergizes with loss of *Ccm2* to induce CCM

**Fig. 5.**
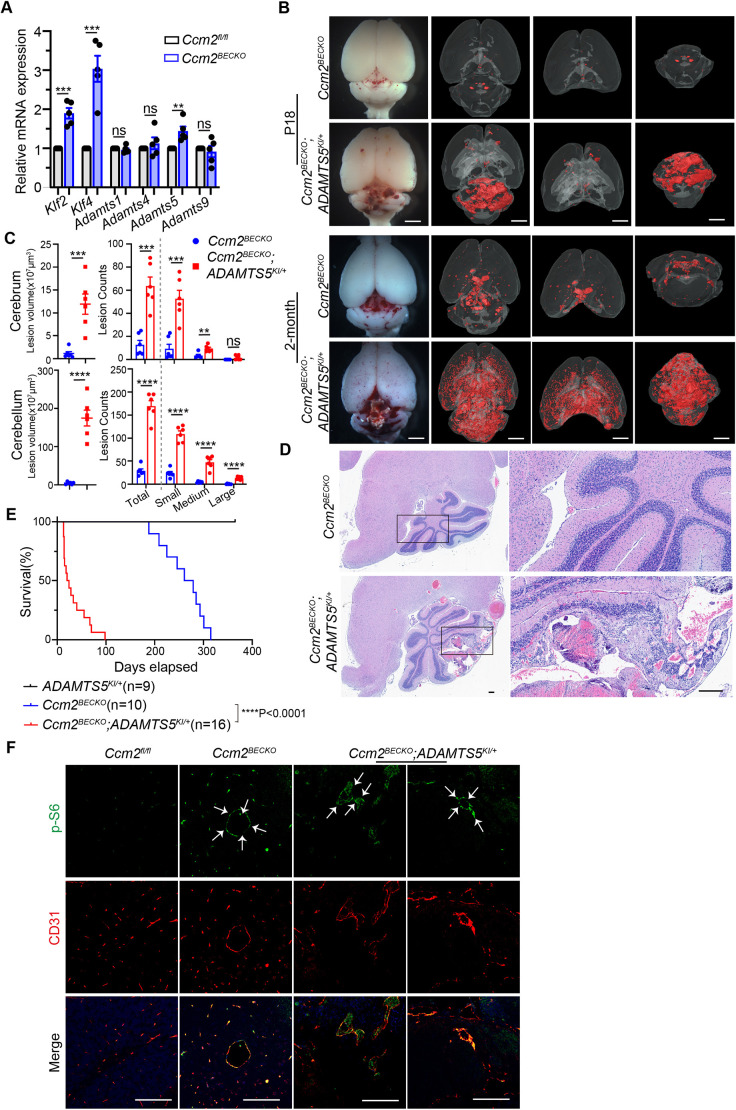
**Expression of ADAMTS5 in brain endothelial cells synergizes with *Ccm2* deficiency to induce CCM lesion formation.** (A) Relative mRNA expression level of CCM downstream genes, *Klf2*, *Klf4*, *Adamts1*, *Adamts4*, *Adamts5* and *Adamts9*, in endothelial cells isolated from control and *Ccm2^BECKO^* mice at P6. *n*≥3 for each group was used. Data are presented as mean±s.e.m. and statistical significance was determined using an unpaired two-tailed Student's *t*-test. ns, not significant; **P<0.01; ***P<0.001. (B) Representative micrographs and micro-CT images of brains from *Ccm2^BECKO^* and *Ccm2^BECKO^;ADAMTS5^KI/+^* mice at P18 and 2 months. Scale bars: 2 mm. (C) Quantification of CCM lesion volumes, total lesion counts (left of the gray dashed line) and the counts of lesions of different size groups (right of the gray dashed line) in the cerebrum and cerebellum of *Ccm2^BECKO^* (*n*=6) and *Ccm2^BECKO^;ADAMTS5^KI/+^* (*n*=6) mice at P18. Small lesions, volume≤10^6^ μm^3^; medium lesions, volume=10^6^-10^7^ μm^3^; large lesions, volume≥10^7^ μm^3^. Data are presented as mean±s.e.m. and statistical significance was determined using an unpaired two-tailed Student's *t*-test. ns, not significant; **P<0.01; ***P<0.001; ****P<0.0001. (D) H&E staining of brain sections of *Ccm2^BECKO^* and *Ccm2^BECKO^;ADAMTS5^KI/+^* mice at P18. Panels on the right show higher-magnification views. Scale bars: 200 μm. (E) Postnatal survive curve of *ADAMTS5^KI/+^* (*n*=9), *Ccm2^BECKO^* (*n*=10) and *Ccm2^BECKO^;ADAMTS5^KI/+^* mice (*n*=16). Statistical analysis was performed using the Mantel–Cox test. ****P<0.0001. (F) Co-immunostaiinng of p-S6 and CD31 showing increased p-S6 in CCM lesions of *Ccm2^BECKO^* and *Ccm2^BECKO^;ADAMTS5^KI/+^* mice compared to that in *Ccm2^fl/fl^* control at P18. White arrows indicate increased p-S6 in CCM lesions in *Ccm2^BECKO^* and *Ccm2^BECKO^;ADAMTS5^KI/+^* mice. Scale bars: 100 μm. Images are representative of *n*=5-6 for each group.

We have previously demonstrated that the BEC Dre-Cre system can be used to generate the chronic CCM mouse model by driving efficient Ccm gene deletion, with *Ccm2* deletion conferring modest lesion burden among the three Ccm genes (Yang et al., 2022). MEKK3–KLF2/4 signaling has been reported to function downstream of CCM signaling for CCM lesion formation (Zhou et al., 2016). Gene expression analysis demonstrated that the expression of *Klf2* and *Klf4* was robustly increased, but the expression of *Adamts5* was modestly increased in cerebellum endothelial cells of P6 *Ccm2^BECKO^* (*Tie2-Dre;Mfsd2a-CrexER;Ccm2^fl/fl^*) mice (Fig. 5A).

To determine whether further increased expression of *ADAMTS5* contributes to CCM formation in a *Ccm2*-deficient background, we generated *Ccm2^BECKO^;ADAMTS5^KI/+^* mice*.* Histological and micro-CT analysis revealed that the *Ccm2^BECKO^;ADAMTS5^KI/+^* mice had markedly increased hemorrhages and CCM lesion burden in comparison to littermate *Ccm2^BECKO^* mice observed visually at P18 and 2 months (Fig. 5B). Micro-CT analysis of CCM lesion burden at P18 showed the significant increase in lesion volume in the cerebrum (10.7-fold) and cerebellum (38.2-fold) (Fig. 5C). The total counts of CCM lesions in *Ccm2^BECKO^;ADAMTS5^KI/+^* mice increased by 5.0- and 5.8-fold in the cerebrum and cerebellum, respectively, in comparison to those of littermate *Ccm2^BECKO^* mice. We divided the CCM lesions into three groups according to the volume (small, ≤10^6^ μm^3^; medium, 10^6^-10^7^ μm^3^; large, ≥10^7^ μm^3^). Quantification of the lesion groups showed that counts of each group were significantly increased (for the cerebrum, small lesions increased from 9.33±9.52 to 52.67±17.81, medium lesions from 3.50±2.25 to 9.50±2.73; and large lesions from 0.0 to 1.50±1.97; for the cerebellum, small lesions increased from 23.67±9.09 to 109.00±16.99, medium lesions from 4.67±1.63 to 47.33±13.71, and large lesions from 0.50±0.83 to 13.30±3.39; values represent mean±s.e.m.). Examination of H&E-stained sections confirmed a significant increase in the count and size of vascular lesions in *Ccm2^BECKO^;ADAMTS5^KI/+^* mice compared with those in littermate *Ccm2^BECKO^* mice (Fig. 5D). Finally, brain endothelial gain of ADAMTS5 combined with *Ccm2* deficiency resulted in the death of 50% of animals by P21, with complete lethality by 3 months of age; in comparison, *Ccm2^BECKO^* mice were all viable up to 7 months of age (Fig. 5E). These results indicate the strong synergy between *Ccm2* loss of function and *ADAMTS5* gain of function to amplify CCM formation.

Gain-of-function variants in phosphatidylinositol 3-kinase (PI3K) have been shown to increase CCM burden in both human patients and mouse models (Weng et al., 2021; Ren et al., 2021; Peyre et al., 2021). CCM and PI3K signaling converge on mTOR activation and phosphorylation of ribosomal protein S6 (p-S6, encoded by *Rps6*), a downstream effector of mTOR signaling (Ren et al., 2021). To determine whether ADAMTS5 expression influences PI3K downstream signaling, we assessed p-S6 levels in cerebellar lysates and isolated endothelial cells. No increase in p-S6 was detected in *Cdh5-CreER^T2^;ADAMTS5^KI/+^* samples compared with controls (Fig. S6A). Consistent with previous reports (Ren et al., 2021), immunostaining was able to detect increased level of p-S6 in vessels of *Ccm2^BECKO^* brains, but no further enhancement of p-S6 level was observed in *Ccm2^BECKO^;ADAMTS5^KI/+^* brains (Fig. 5F). These findings suggest that ADAMTS5-mediated exacerbation of CCM lesion burden occurs independently of PI3K–mTOR signaling.

**Fig. 6.**
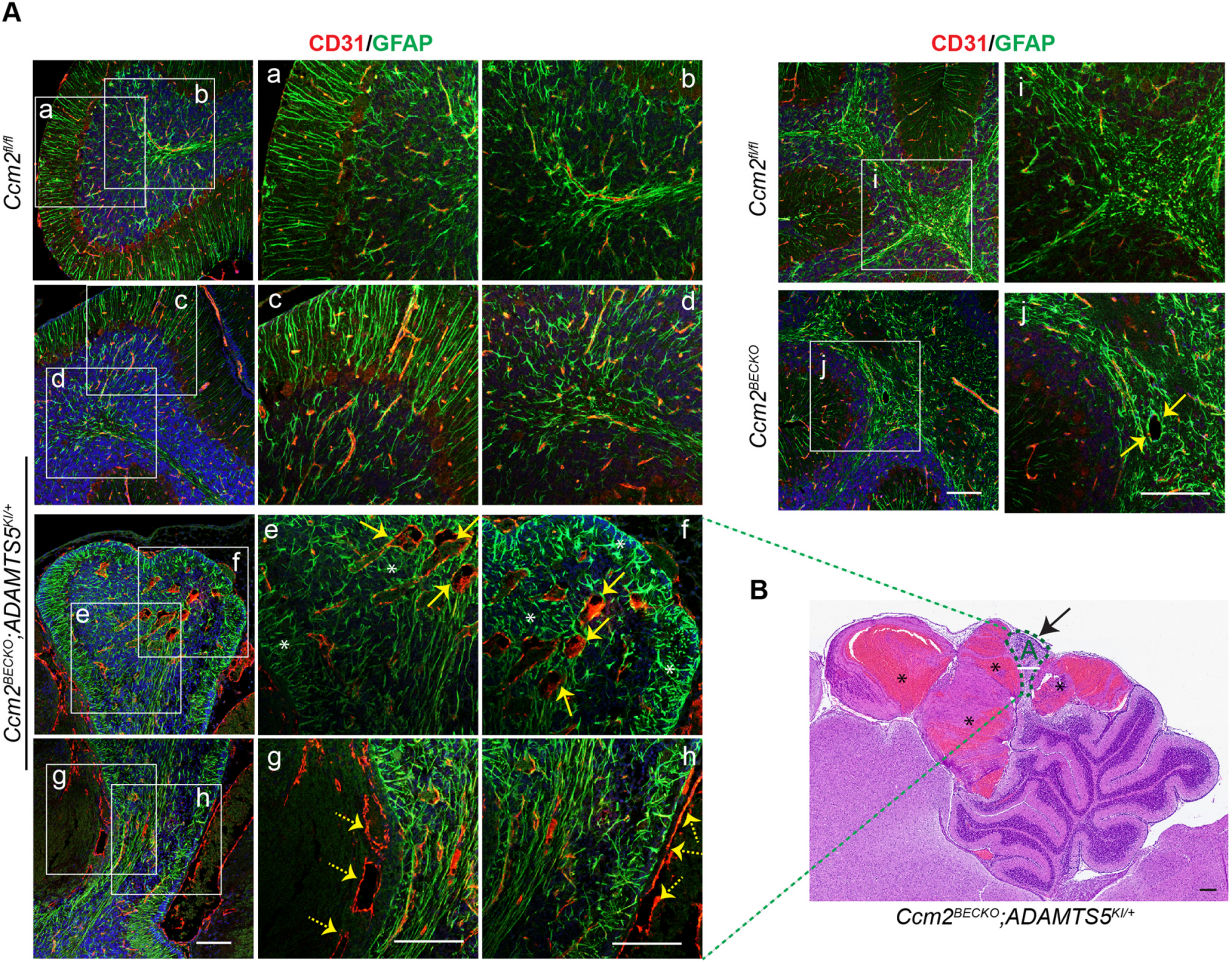
**Abnormal astrocyte arrangement at the CCM lesion region in *Ccm2^BECKO^* and *Ccm2^BECKO^;ADAMTS5^KI/+^* mice.** (A) Co-immunostaining of GFAP and CD31 showing disorganized astrocytes in the brain region with small CCM lesions (panels e,f,j, yellow solid arrows) in *Ccm2^BECKO^* and *Ccm2^BECKO^;ADAMTS5^KI/+^* mice, and reduced GFAP and CD31 signal overlap in vessels of small lesions and absence of astrocyte overlap with the endothelium of large CCM lesions (panels g,h; yellow dashed arrows) in *Ccm2^BECKO^;ADAMTS5^KI/+^* mice. White asterisks indicate disordered astrocytes in the cerebellum. Normal parallel GFAP (astrocytes) arrangement was observed in both *Ccm2^fl/fl^* (panels a,b) and non-CCM lesion brain regions (panels c,d) in *Ccm2^BECKO^;ADAMTS5^KI/+^* mice. Nuclear staining is shown in blue. Scale bars: 200 μm. (B) H&E staining of an adjacent section indicates the orientation of the immunostained region, lesion size and location in the cerebellum. The black arrow indicates small CCM lesions, and black asterisks indicate large CCM lesions, the locations of which were consistent with GFAP/CD31 staining (panels e-h). Horizontal lines denote the regions corresponding to the areas where panels e,f, and g,h are located. Scale bar: 200 μm. Images are representative of *n*=3-4 for each group.

Interactions between brain endothelial cells and surrounding astrocytes and inflammatory cells have been shown to influence CCM lesion severity (Lopez-Ramirez et al., 2021; Lai et al., 2022; Onyeogaziri et al., 2024). To evaluate whether ADAMTS5 expression alters the neurovascular microenvironment, we examined ECM composition and mural cell coverage. In the induced *Cdh5-CreER^T2^;ADAMTS5^KI/+^* and control brains, co-immnunostaining of collagen IV and CD31 revealed no differences in vascular integrity, with both groups showing effective colocalization (Fig. S6B). Similarly, no significant difference were observed between *ADAMTS5^BECKI^* and littermate control mice (Fig. S6C) or between *Ccm2^BECKO^;ADAMTS5^KI/+^* and littermate control mice (Fig. S6D). In contrast, GFAP immunostaining revealed a disorganized astrocytic pattern in regions containing CCM lesions in *Ccm2^BECKO^;ADAMTS5^KI/+^* brains. The GFAP signal was largely absent adjacent to endothelial cells within large lesions and showed reduced overlap with CD31 in smaller lesions. No aberrant GFAP patterning was observed in non-lesion regions of *Ccm2^BECKO^;ADAMTS5^KI/+^* compared with *Ccm2^BECKO^* brains (Fig. 6). These data suggest that ADAMTS5 expression exacerbates CCM lesions by altering endothelial interactions with other cell types in the neurovascular unit or by modifying ECM composition within the lesion environment.

## DISCUSSION

In this study, we systematically elucidated the regulatory roles of ADAMTS5 in cardiovascular development and the pathogenesis of CCM through a human *ADAMTS5* knockin mouse model. The results demonstrated that sustained endothelial ADAMTS5 expression leads to premature degradation of cardiac jelly and aberrant breakdown of proteoglycans in endocardial cushions, ultimately resulting in embryonic lethality. Postnatal induction of ADAMTS5 overexpression in endothelial cells resulted in valvular developmental defects. Although brain endothelial-specific sustained ADAMTS5 expression did not induce obvious vascular malformations, it markedly exacerbated CCM lesions and shortened survival in *Ccm2*-deficient mice. These findings establish that precise regulation of ADAMTS5 expression is essential for cardiovascular development and homeostasis, and highlight its synergistic pathogenic effect with the CCM pathway.

As a critical structure in embryonic heart development, cardiac jelly sustains homeostasis via balanced proteoglycan synthesis and degradation (Hinton et al., 2006; Lockhart et al., 2011). Our results showed that ADAMTS5 overexpression disrupts the structural integrity of cardiac jelly through excessive degradation, thereby impairing ventricular trabeculation and atrioventricular cushion remodeling. This supports previous findings that elevated ADAMTS4 and ADAMTS5 activity may impair normal cardiac jelly formation (Zhou et al., 2015). Interestingly, although ADAMTS5 deficiency leads to aortic valve calcification and vascular wall structural abnormalities, our data revealed that its overexpression also induces severe valvular malformation. Together, these findings emphasize that the dynamic balance in ADAMTS5 enzymatic activity is crucial for normal cardiovascular development (Santamaria and de Groot, 2020; Barallobre-Barreiro et al., 2021). Physiological levels of ADAMTS5 support tissue remodeling, but abnormal expression results in an ECM imbalance. This bidirectional regulation provides new insights into the pathogenesis of congenital cardiac valve diseases. Comparison with other ADAMTS family members further underscores this point: ADAMTS9 deficiency leads to aortic valve abnormalities and versican accumulation (Kern et al., 2010), whereas ADAMTS16 deficiency results in bicuspid aortic valve formation (Lin et al., 2024). The studies show that ADAMTS5 regulates a broader spectrum of processes, including cardiac jelly homeostasis, valvulogenesis and vascular integrity (Zhou et al., 2015; Li et al., 2017; Zhu et al., 2025).

The CCM pathway maintains vascular stability through the CCM1–CCM2–CCM3 complex, which regulates the MEKK3–KLF2/4–ADAMTS axis (Zhou et al., 2016). We found that ADAMTS5 overexpression strongly synergizes with *Ccm2* deficiency to enhance the aggressiveness of CCM lesions, potentially through the influence of versican degradation products on vascular endothelial stability. This aligns with previous work showing that *Adamts5* deficiency suppresses CCM lesion formation in *Ccm1*-deficient models, confirming that *Adamts5* acts as a downstream effector of the CCM pathway (Hong et al., 2020). Importantly, induction of ADAMTS5 expression alone in brain endothelial cells was insufficient to trigger CCM, indicating that its pathogenic effects depend on dysfunction of the CCM complex, providing a theoretical basis for selective therapeutic targeting. Despite the well-established role of gain-of-function variants in PI3K in elevating CCM burden, our findings indicate that ADAMTS5-mediated exacerbation of CCM lesion burden is independent of PI3K–mTOR signaling at the level of p-S6. This suggests that ADAMTS5 acts through alternative pathways to influence CCM development. Despite the overexpression of ADAMTS5, our results show no distribution and density changes between endothelial cells and their basement membrane. On the contrary, the disordered astrocytic pattern demonstrated by GFAP immunostaining indicates that ADAMTS5 exerts its effect by interfering with the communication between endothelial cells and astrocytes. These two cell types are vital constituents of the neurovascular unit.

As a major aggrecan-degrading enzyme, ADAMTS5 is well known for driving cartilage destruction in osteoarthritis (Wang et al., 2024; Glasson et al., 2005; Stanton et al., 2005). The regulatory mechanism of ADAMTS5 on matrix proteoglycans identified in this study offers clues for understanding the pathogenesis of osteoarthritis and cardiovascular diseases. The ability of ADAMTS5 to both degrade versican in the cardiovascular system and cleave aggrecan in joints depends on its protease activity, suggesting that ADAMTS5-targeted inhibitors could simultaneously modulate matrix homeostasis in multiple tissues (Clement-Lacroix et al., 2022). The development of ADAMTS5 inhibitors for osteoarthritis treatment needs careful consideration of potential cardiovascular effects, including the risk of vascular calcification or valvular abnormalities (Larkin et al., 2015). The humanized ADAMTS5 mouse model generated in this study provides a platform to evaluate both the efficiency and systemic safety of ADAMTS5 modulators. ADAMTS5-mediated proteoglycan degradation products may serve as shared biomarkers for monitoring the progression of cardiovascular diseases and osteoarthritis.

Previous studies have largely focused on *Adamts5* deficiency phenotypes in the cardiovascular system. Our study demonstrated the pathological consequences of ADAMTS5 overexpression in a humanized model, underscoring the necessity for strict regulation of its activity. In the context of CCM, this study revealed the synergistic role of ADAMTS5 expression and *Ccm2* deficiency in CCM pathogenesis, enhancing our understanding of the molecular mechanism of CCM. Nonetheless, the contribution of ADAMTS5 to other proteoglycan targets and its role in adult cardiovascular homeostasis and osteoarthritis progression remain to be clarified. Potential species-specific interactions between human ADAMTS5 and the mouse proteoglycan environment should also be considered.

Taken together, our findings identify ADAMTS5 as a potential therapeutic target for CCM progression. Strategies targeting ADAMTS5 in CCM or osteoarthritis must be carefully designed to achieve cell-type specificity. The humanized ADAMTS5 knockin model described here provides a valuable tool for screening modulators with both osteoarthritis efficacy and cardiovascular safety.

## MATERIALS AND METHODS

### Mice

*Tie2-Cre*, *Nfatc1-Cre*, *Cdh5-CreER^T2^*, *Tie2-Dre*, *Mfsd2a-CrexER* and *Ccm2^fl/fl^* animals have been described previously (Zhou et al., 2015; Yang et al., 2022, 2023). The *Ccm2^BECKO^* (*Tie2-Dre;Mfsd2a-CrexER;Ccm2^fl/fl^*) mice were described in Yang et al. (2022). *ADAMTS5^KI^* mice were generated via embryonic stem cell-based gene targeting by Shanghai Biomodel Organism Science and Technology Development. Experimental animals were maintained on a 129/C57BL/6J mixed genetic background. Both male and female mice were used in the experiments. Littermates were used as controls for all experiments.

The primers used to genotype *ADAMTS5^KI^* mice were as follows: ADAMTS5KI-Forward, 5ʹ-AAGGGGGAGGATTGGGAAGACA-3ʹ, and ADAMTS5KI-Reverse, 5ʹ-CAGAAGGAGCGGGAGAAATGGATA-3ʹ.

### Study approval

All animal ethics and protocols were approved by the Institutional Animal Care and Use Committee of Tianjin Medical University.

### Mouse embryo processing

All E9.5-E12.5 embryos were dissected and then imaged under a stereomicroscope (Nikon, SMZ18). The embryos were fixed in 4% polyformaldehyde (PFA) at 4°C overnight and subjected to the next protocols.

### Induction of *ADAMTS5* expression *in vivo*

4-hydroxytamoxifen (Sigma-Aldrich, H7904) was dissolved in 1% ethanol-corn oil (0.5 mg/ml, C8267, Sigma-Aldrich) and used to induce *Cdh5-CreER^T2^;ADAMTS5^KI/+^* mice for sustained expression of the *ADAMTS5* gene. P1 pups were intragastrically injected with a single dose of 75 μl (37.5 μg) of 4-hydroxytamoxifen once.

### Histological analysis

Fresh mouse embryos and all tissues were fixed in 4% PFA overnight and paraffin-embedded after gradual dehydration in 100% ethanol. Paraffin sections (7 μm) were obtained using a microtome (RM2245, Leica) were stained with H&E and Alcian Blue using standard protocols. Immunostaining and immunohistochemistry protocols have been previously described (Yang et al., 2022). Briefly, deparaffinized sections underwent successive processes consisting of rehydration with xylene, antigen retrieval, blocking in normal donkey serum with BSA and PBS containing 0.1% Tween-20 (Solarbio), and incubation with primary antibodies overnight at 4°C. The sections were then incubated with the secondary antibody for 2 h at room temperature after washing, and then imaged using the Nikon microscope (Ni-U). Trabeculae myocardium and compacted myocardium thickness and cardiac jelly area were quantified by ImageJ. Quantitative data of trabeculae myocardium and compacted myocardium thickness, and cardiac jelly area are from three sections for each point in E9.5, E10.5 and E11.5 embryos. Representative images from at least three or more independent experiments are shown.

### Vibratome sections and staining

Fresh brain tissue was fixed in 4% PFA for 2-4 h and embedded in low-melting agarose. The coronal vibratome section was sliced in units of 100 μm using a Leica VT1200s vibratome. After the penetration of PBS solution containing 0.3% Triton X-100 and BSA, the sections were washed with PBS and stained with the antibody. Images were acquired using an Axio-Imager LSM-800 confocal microscope (Carl Zeiss).

### Isolation of cerebellum endothelial cells

The cerebellum was removed from mice after they were anesthetized with 0.2% avertin (Sigma-Aldrich) and perfused with cold PBS. The cerebellum was digested with digestion mix [Dulbecco's modified Eagle medium (DMEM; Corning) containing 10% fetal bovine serum (FBS, Corning), 1 mg/ml collagenase/dispase (Roche, 10269638001) and benzonase (Millipore, E1014, 1:2000 dilution)] at 37°C for 15 min until complete digestion. The single-cell suspension was incubated with anti-CD31-conjugated magnetic beads (Miltenyi Biotec, 130-097-418) at 4°C for 15 min after they were passed through a 70 μm strainer. We collected microbead-bound cells using MACS MS columns (MiltenyiBiotec) for subsequent qPCR analysis.

### Western blot analysis

Whole tissue was added to the lysis buffer (150 mM NaCl, 1% NP-40 and 50 mM Tris-HCl, pH 8.0) with protease and phosphatase inhibitors. Protein supernatants were harvested after 12,000 ***g*** centrifugation for 20 min at 4°C. The BCA kit (Thermo Fisher Scientific, 23252) was used to quantify protein concentration. 20 μg protein samples were separated by SDS-PAGE within the running buffer and transferred to nitrocellulose membranes within membrane transfer solution. Membranes were blocked with 5% skim milk at room temperature and incubated with primary antibodies at 4°C overnight. The next day, membranes were washed four times with PBS containing 0.1% Tween-20 (Solarbio) and then incubated with the secondary antibody at room temperature for 1 h. Membranes were prepared for exposure to acquire images using ECL chemiluminescence solution (Thermo Fisher Scientific) incubation.

### Antibodies and reagents

#### Immunofluorescence

Primary antibodies and reagents used for immunofluorescence were as follows: Dylight 594 isolectin B4 (Vector Laboratories, DL1207, RRID:AB_2336415, 1:200 dilution), anti-endomucin (Abcam, ab106100, RRID:AB_10859306, 1:200 dilution), MF20 antibody (Developmental Studies Hybridoma Bank, AB2147781, RRID:AB_2147781, 1:200 dilution), rat anti-CD31 (Dianova, DIA-310, RRID:AB_2631039, 1:300 dilution), mouse anti-SMA (Sigma-Aldrich, A2547, RRID:AB_476701, 1:500 dilution), rabbit anti-GFAP (Cell Signaling Technology, 80788, RRID:AB_2799963, 1:200 dilution), rabbit anti-collagen IV (Abcam, ab6586, RRID:AB_305584, 1:200 dilution), anti-versican (Abcam, ab270445, RRID:AB_3714692, 1:200 dilution), anti-GFP (Cell Signaling Technology, 2555s, RRID:AB_10692764, 1:500 dilution), anti-p-S6 (Ser 235/236) ribosomal protein (Cell Signaling Technology, 4858T, RRID:AB_916156, 1:200 dilution), anti-prosurfactant protein C (SPC) (Abcam, ab211326, RRID:AB_2927746, 1:200 dilution) and anti-PCNA (Cell Signaling Technology, 2586s, RRID:AB_2160343, 1:200 dilution).

The secondary antibodies used were as follows: ImmPRESS (peroxidase) secondary antibody (goat anti-rat, Vector Laboratories, MP-7444, RRID:AB_2336530) and TSA fluorescence system working solution (PerkinElmer, TSA-plus tetramethylrhodamine System, NEL742001KT) for CD31 immunostaining, and anti-mouse IgG (H+L), F(ab’)2 Fragment Alexa Fluor 488 conjugate (Cell Signaling Technology, 4408, RRID:AB_10694704, 1:500 dilution), anti–mouse IgG (H+L) Alexa Fluor 594 conjugate (Cell Signaling Technology, 8890, RRID:AB_2714182, 1:500 dilution), anti-rabbit IgG (H+L) Alexa Fluor 594 conjugate (Cell Signaling Technology, 8889, RRID:AB_2716249, 1:500 dilution) and anti-rat IgG (H+L) Alexa Fluor 488 conjugate (Cell Signaling Technology, 4416, RRID:AB_10693769, 1:500 dilution).

#### Immunohistochemistry

The antibodies and reagents used for immunohistochemistry were anti-versican (Abcam, ab270445, RRID:AB_3714692, 1:200 dilution), HRP-conjugated goat anti-rabbit IgG (H+L) (Proteintech, SA00001-2, RRID:AB_2722564, 1:2000 dilution) and a rabbit-specific IHC polymer detection kit HRP/DAB (Abcam, ab209101).

#### Vibratome staining

Dylight 594 Isolectin B4 (Vector Laboratories, DL1207, RRID:AB_2336415, 1:200 dilution) was used for staining vibratome sections.

#### Western blotting

The antibodies used for western blotting were anti-GFP (Cell Signaling Technology, 2555s, RRID:AB_10692764, 1:500 dilution), anti-p-S6 (Ser 235/236) ribosomal protein (Cell Signaling Technology, 4858T, RRID:AB_916156, 1:200 dilution) and anti-ACTB (Abclonal, AC026, RRID:AB_2768234, 1:50,000 dilution).

### Real-time PCR analysis

TRIzol reagents (Thermo Fisher Scientific, 15596018) were used to extract total RNA. The StarScript II First-strand cDNA Synthesis Kit (Yeason, 11141ES) was used to produce cDNA. ChamQ Universal SYBR qPCR Master Mix (Vazyme Biotech Co, Q711-03) was added to the system for real-time qPCR experiments. Real-time PCR was performed on QuantStudio 5 systems (Applied Biosystem, A28139).

The following are the primers used in this study: h-*GAPDH*-Forward, 5ʹ-GAGTCAACGGATTTGGTCGT-3ʹ; h-*GAPDH*-Reverse, 5ʹ-GATCTCGCTCCTGGAAGATG-3ʹ; h-*ADAMTS5*-Forward, 5ʹ-CTGTGCTGTGATTGAAGACGA-3ʹ; h-*ADAMTS5*-Reverse, 5ʹ-CAGGATCTGCTTTCGTGGTAG-3ʹ; m-*Gapdh*-Forward, 5ʹ-GTCCCGTAGACAAAATGGTGA-3ʹ; m-*Gapdh*-Reverse, 5ʹ-TTTGATGTTAGTGGGGTCTCG-3ʹ; m-*Klf2*-Forward, 5ʹ-CGCCTCGGGTTCATTTC-3ʹ; m-*Klf2*-Reverse, 5ʹ-AGCCTATCTTGCCGTCCTTT-3ʹ; m-*Klf4*-Forward, 5ʹ-GTGCCCCGACTAACCGTTG-3ʹ; m-*Klf4*-Reverse, 5ʹ-GTCGTTGAACTCCTCGGTCT-3ʹ; m-*Adamts1*-Forward, 5ʹ-CCTTACGGCAGCAGACACA-3ʹ; m-*Adamts1*-Reverse, 5ʹ-AATCTGCTGTCAGTGGCCC-3ʹ; m-*Adamts4*-Forward, 5ʹ-CAGTGCCCGATTCATCACT-3ʹ; m-*Adamts4*-Reverse, 5ʹ-GAGTCAGGACCGAAGGTCAG-3ʹ; m-*Adamts5*-Forward, 5ʹ-CGACCCTCAAGAACTTTTGC-3ʹ; m-*Adamts5*-Reverse, 5ʹ-CGTCATGAGAAAGGCCAAGT-3ʹ; m-*Adamts9*-Forward, 5ʹ-AGCGGAAAATCAGAATGCGAAAA-3ʹ; and m-*Adamts9*-Reverse, 5ʹ-TGAAGGTTTGCTCCGTGGTATAA-3ʹ.

### Contrast-enhanced X-ray micro-CT and analysis

The brain was harvested and fixed in 4% PFA after the mice were anesthetized and perfused with 4% PFA/PBS solution. The brains were immersed in Lugol solution (Sigma-Aldrich, L6146) for 48 h and scanned using the micro-CT system (MicroXCT-400, Xradia, CA, USA). All samples were randomly scanned by a masked operator. The images were acquired with the following parameters: 50 kV, 10 W, 721 projections, 3 s integration per 180° rotation.

Post-lesion labeling of micro-CT scanning results was done by a masked investigator who was unaware of experimental details. We used Avizo 3D software (Thermo Fisher Scientific) to analyze the data and the specific protocols as previously described (Yang et al., 2023).

### Statistical analysis

All tables were analyzed with χ^2^ analysis, and survival curves were analyzed with the Mantel–Cox test. All other statistical analyses in the study were performed with an unpaired two-tailed *t*-test using GraphPad Prism 8.0 statistical software. Statistical significance was considered when *P*<0.05.

## Supplementary Material

10.1242/dmm.052668_sup1 Supplementary information

## References

[DMM052668C1] Barallobre-Barreiro, J., Radovits, T., Fava, M., Mayr, U., Lin, W. Y., Ermolaeva, E., Martinez-Lopez, D., Lindberg, E. L., Duregotti, E., Daroczi, L. et al. (2021). Extracellular matrix in heart failure: role of ADAMTS5 in proteoglycan remodeling. *Circulation* 144, 2021-2034. 10.1161/CIRCULATIONAHA.121.055732 34806902 PMC8687617

[DMM052668C2] Cikach, F. S., Koch, C. D., Mead, T. J., Galatioto, J., Willard, B. B., Emerton, K. B., Eagleton, M. J., Blackstone, E. H., Ramirez, F., Roselli, E. E. et al. (2018). Massive aggrecan and versican accumulation in thoracic aortic aneurysm and dissection. *JCI Insight* 3, e97167. 10.1172/jci.insight.97167 29515038 PMC5922288

[DMM052668C3] Clement-Lacroix, P., Little, C. B., Smith, M. M., Cottereaux, C., Merciris, D., Meurisse, S., Mollat, P., Touitou, R., Brebion, F., Gosmini, R. et al. (2022). Pharmacological characterization of GLPG1972/S201086, a potent and selective small-molecule inhibitor of ADAMTS5. *Osteoarthritis Cartilage* 30, 291-301. 10.1016/j.joca.2021.08.012 34626798

[DMM052668C4] Dupuis, L. E., McCulloch, D. R., McGarity, J. D., Bahan, A., Wessels, A., Weber, D., Diminich, A. M., Nelson, C. M., Apte, S. S. and Kern, C. B. (2011). Altered versican cleavage in ADAMTS5 deficient mice; a novel etiology of myxomatous valve disease. *Dev. Biol.* 357, 152-164. 10.1016/j.ydbio.2011.06.041 21749862 PMC4435578

[DMM052668C5] Dupuis, L. E., Nelson, E. L., Hozik, B., Porto, S. C., Rogers-DeCotes, A., Fosang, A. and Kern, C. B. (2019). Adamts5(-/-) mice exhibit altered aggrecan proteolytic profiles that correlate with ascending aortic anomalies. *Arterioscler. Thromb. Vasc. Biol.* 39, 2067-2081. 10.1161/ATVBAHA.119.313077 31366218 PMC6761016

[DMM052668C6] Fava, M., Barallobre-Barreiro, J., Mayr, U., Lu, R., Didangelos, A., Baig, F., Lynch, M., Catibog, N., Joshi, A., Barwari, T. et al. (2018). Role of ADAMTS-5 in aortic dilatation and extracellular matrix remodeling. *Arterioscler. Thromb. Vasc. Biol.* 38, 1537-1548. 10.1161/ATVBAHA.117.310562 29622560 PMC6026471

[DMM052668C7] Glasson, S. S., Askew, R., Sheppard, B., Carito, B., Blanchet, T., Ma, H. L., Flannery, C. R., Peluso, D., Kanki, K., Yang, Z. et al. (2005). Deletion of active ADAMTS5 prevents cartilage degradation in a murine model of osteoarthritis. *Nature* 434, 644-648. 10.1038/nature03369 15800624

[DMM052668C8] Hinton, R. B., Jr, Lincoln, J., Deutsch, G. H., Osinska, H., Manning, P. B., Benson, D. W. and Yutzey, K. E. (2006). Extracellular matrix remodeling and organization in developing and diseased aortic valves. *Circ. Res.* 98, 1431-1438. 10.1161/01.RES.0000224114.65109.4e 16645142

[DMM052668C9] Hong, C. C., Tang, A. T., Detter, M. R., Choi, J. P., Wang, R., Yang, X., Guerrero, A. A., Wittig, C. F., Hobson, N., Girard, R. et al. (2020). Cerebral cavernous malformations are driven by ADAMTS5 proteolysis of versican. *J. Exp. Med.* 217, e20200140. 10.1084/jem.20200140 32648916 PMC7537394

[DMM052668C10] Kelwick, R., Desanlis, I., Wheeler, G. N. and Edwards, D. R. (2015). The ADAMTS (A Disintegrin and Metalloproteinase with Thrombospondin motifs) family. *Genome Biol.* 16, 113. 10.1186/s13059-015-0676-3 26025392 PMC4448532

[DMM052668C11] Kern, C. B., Wessels, A., McGarity, J., Dixon, L. J., Alston, E., Argraves, W. S., Geeting, D., Nelson, C. M., Menick, D. R. and Apte, S. S. (2010). Reduced versican cleavage due to Adamts9 haploinsufficiency is associated with cardiac and aortic anomalies. *Matrix Biol.* 29, 304-316. 10.1016/j.matbio.2010.01.005 20096780 PMC2862783

[DMM052668C12] Lai, C. C., Nelsen, B., Frias-Anaya, E., Gallego-Gutierrez, H., Orecchioni, M., Herrera, V., Ortiz, E., Sun, H., Mesarwi, O. A., Ley, K. et al. (2022). Neuroinflammation plays a critical role in cerebral cavernous malformation disease. *Circ. Res.* 131, 909-925. 10.1161/CIRCRESAHA.122.321129 36285625 PMC9669201

[DMM052668C13] Larkin, J., Lohr, T. A., Elefante, L., Shearin, J., Matico, R., Su, J. L., Xue, Y., Liu, F., Genell, C., Miller, R. E. et al. (2015). Translational development of an ADAMTS-5 antibody for osteoarthritis disease modification. *Osteoarthritis Cartilage* 23, 1254-1266. 10.1016/j.joca.2015.02.778 25800415 PMC4516626

[DMM052668C14] Li, F., Song, R., Ao, L., Reece, T. B., Cleveland, J. C., Jr, Dong, N., Fullerton, D. A. and Meng, X. (2017). ADAMTS5 Deficiency in calcified aortic valves is associated with elevated pro-osteogenic activity in valvular interstitial cells. *Arterioscler. Thromb. Vasc. Biol.* 37, 1339-1351. 10.1161/ATVBAHA.117.309021 28546218 PMC5516888

[DMM052668C15] Lin, Y., Yang, Q., Lin, X., Liu, X., Qian, Y., Xu, D., Cao, N., Han, X., Zhu, Y., Hu, W. et al. (2024). Extracellular matrix disorganization caused by ADAMTS16 deficiency leads to bicuspid aortic valve with raphe formation. *Circulation* 149, 605-626. 10.1161/CIRCULATIONAHA.123.065458 38018454

[DMM052668C16] Lockhart, M., Wirrig, E., Phelps, A. and Wessels, A. (2011). Extracellular matrix and heart development. *Birth Defects Res. A Clin. Mol. Teratol* 91, 535-550. 10.1002/bdra.20810 21618406 PMC3144859

[DMM052668C17] Lopez-Ramirez, M. A., Lai, C. C., Soliman, S. I., Hale, P., Pham, A., Estrada, E. J., McCurdy, S., Girard, R., Verma, R., Moore, T. et al. (2021). Astrocytes propel neurovascular dysfunction during cerebral cavernous malformation lesion formation. *J. Clin. Investig.* 131, e20200140. 10.1172/JCI139570 PMC8245174 34043589

[DMM052668C18] Männer, J. and Yelbuz, T. M. (2019). Functional morphology of the cardiac jelly in the tubular heart of vertebrate embryos. *J. Cardiovasc. Dev. Dis.* 6, 12. 10.3390/jcdd6010012 30818886 PMC6463132

[DMM052668C19] Onyeogaziri, F. C., Smith, R., Arce, M., Huang, H., Erzar, I., Rorsman, C., Malinverno, M., Orsenigo, F., Sundell, V., Fernando, D. et al. (2024). Pharmacological blocking of neutrophil extracellular traps attenuates immunothrombosis and neuroinflammation in cerebral cavernous malformation. *Nat. Cardiovasc. Res.* 3, 1549-1567. 10.1038/s44161-024-00577-y 39632986 PMC11634782

[DMM052668C20] Peyre, M., Miyagishima, D., Bielle, F., Chapon, F., Sierant, M., Venot, Q., Lerond, J., Marijon, P., Abi-Jaoude, S., Le Van, T. et al. (2021). Somatic PIK3CA mutations in sporadic cerebral cavernous malformations. *N. Engl. J. Med.* 385, 996-1004. 10.1056/NEJMoa2100440 34496175 PMC8606022

[DMM052668C21] Ren, A. A., Snellings, D. A., Su, Y. S., Hong, C. C., Castro, M., Tang, A. T., Detter, M. R., Hobson, N., Girard, R., Romanos, S. et al. (2021). PIK3CA and CCM mutations fuel cavernomas through a cancer-like mechanism. *Nature* 594, 271-276. 10.1038/s41586-021-03562-8 33910229 PMC8626098

[DMM052668C22] Santamaria, S. and de Groot, R. (2020). ADAMTS proteases in cardiovascular physiology and disease. *Open Biol.* 10, 200333. 10.1098/rsob.200333 33352066 PMC7776578

[DMM052668C23] Stanton, H., Melrose, J., Little, C. B. and Fosang, A. J. (2011). Proteoglycan degradation by the ADAMTS family of proteinases. *Biochim. Biophys. Acta* 1812, 1616-1629. 10.1016/j.bbadis.2011.08.009 21914474

[DMM052668C24] Stanton, H., Rogerson, F. M., East, C. J., Golub, S. B., Lawlor, K. E., Meeker, C. T., Little, C. B., Last, K., Farmer, P. J., Campbell, I. K. et al. (2005). ADAMTS5 is the major aggrecanase in mouse cartilage in vivo and in vitro. *Nature* 434, 648-652. 10.1038/nature03417 15800625

[DMM052668C25] Wang, Z., Shi, W., Wu, L., Xiao, Y., Wang, M., Zhang, S., Chen, Z., Yin, G., Xie, X., Bi, S. et al. (2024). TMF inhibits extracellular matrix degradation by regulating the C/EBPβ/ADAMTS5 signaling pathway in osteoarthritis. *Biomed. Pharmacother.* 174, 116501. 10.1016/j.biopha.2024.116501 38554527

[DMM052668C26] Weng, J., Yang, Y., Song, D., Huo, R., Li, H., Chen, Y., Nam, Y., Zhou, Q., Jiao, Y., Fu, W. et al. (2021). Somatic MAP3K3 mutation defines a subclass of cerebral cavernous malformation. *Am. J. Hum. Genet.* 108, 942-950. 10.1016/j.ajhg.2021.04.005 33891857 PMC8206158

[DMM052668C27] Yang, X., Dai, Z., Gao, C., Yin, Y., Shi, C., Liu, R., Zhuge, Q., Huang, Y., Zhou, B., Han, Z. et al. (2022). Cerebral cavernous malformation development in chronic mouse models driven by dual recombinases induced gene deletion in brain endothelial cells. *J. Cereb. Blood Flow Metab.* 42, 2230-2244. 10.1177/0271678X221105995 35686705 PMC9669998

[DMM052668C28] Yang, X., Wu, S. T., Gao, R., Wang, R., Wang, Y., Dong, Z., Wang, L., Qi, C., Wang, X., Schmitz, M. L. et al. (2023). Release of STK24/25 suppression on MEKK3 signaling in endothelial cells confers cerebral cavernous malformation. *JCI Insight* 8, e160372. 10.1172/jci.insight.160372 36692953 PMC10077477

[DMM052668C29] Zhao, T., Huang, Y., Zhu, J., Qin, Y., Wu, H., Yu, J., Zhai, Q., Li, S., Qin, X., Wang, D. et al. (2025). Extracellular matrix signaling cues: biological functions, diseases, and therapeutic targets. *MedComm (2020)* 6, e70281. 10.1002/mco2.70281 40686923 PMC12271642

[DMM052668C30] Zhou, Z., Rawnsley, D. R., Goddard, L. M., Pan, W., Cao, X. J., Jakus, Z., Zheng, H., Yang, J., Arthur, J. S., Whitehead, K. J. et al. (2015). The cerebral cavernous malformation pathway controls cardiac development via regulation of endocardial MEKK3 signaling and KLF expression. *Dev. Cell* 32, 168-180. 10.1016/j.devcel.2014.12.009 25625206 PMC4589864

[DMM052668C31] Zhou, Z., Tang, A. T., Wong, W. Y., Bamezai, S., Goddard, L. M., Shenkar, R., Zhou, S., Yang, J., Wright, A. C., Foley, M. et al. (2016). Cerebral cavernous malformations arise from endothelial gain of MEKK3-KLF2/4 signalling. *Nature* 532, 122-126. 10.1038/nature17178 27027284 PMC4864035

[DMM052668C32] Zhu, Z., Liu, H., Feng, L., Lu, L., Zhu, J., Liang, Q., Lan, Z., Ye, Y., Wang, S., Chen, A. et al. (2025). Loss of ADAMTS5 promotes vascular calcification via versican/integrin beta1/FAK signal. *Atherosclerosis* 404, 119190. 10.1016/j.atherosclerosis.2025.119190 40215897

