# Supplementary material for: Knockin expression of human ADAMTS5 impairs cardiovascular development and aggravates cerebral cavernous malformations in mice
Source: Dis Model Mech. 2026 Jun 1;19(5):dmm052668. doi: 10.1242/dmm.052668 (PMC13312918; doi:10.1242/dmm.052668)
Supplement: Supplementary information [file dmm-19-052668-s1.pdf]

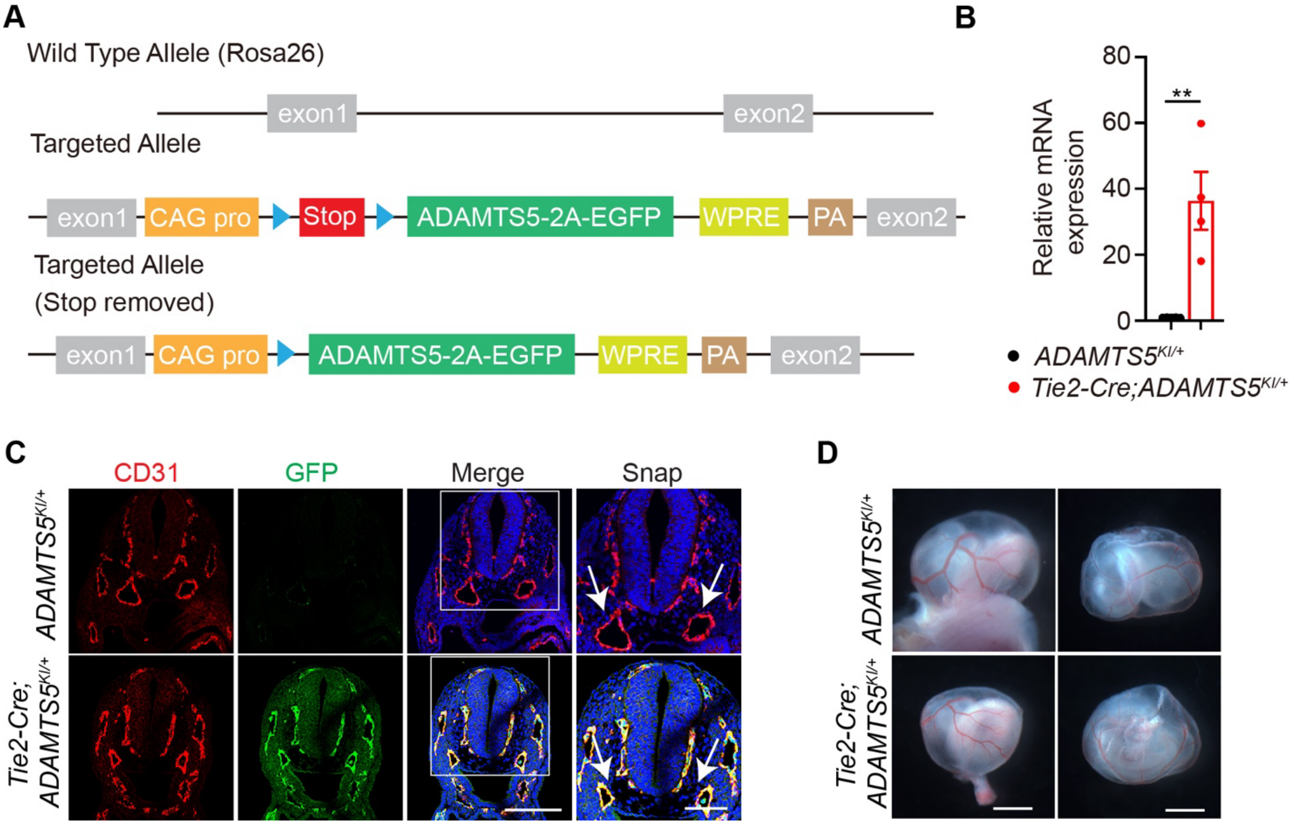

Fig. S1. Generation of the Human-*ADAMTS5* knockin allele and validation of endothelial ADAMTS5 expression. A) Schematic illustration of the *ADAMTS5* knockin (*ADAMTS5^{KI}*) strategy. A LoxP flanked Stop cassette followed by the coding sequence for human ADAMTS5 and 2A-EGFP were inserted in intron 1 of the *Rosa26* gene for conditional expression of human ADAMTS5 in mice. **B)** Relative mRNA expression level of *ADAMTS5* in *ADAMTS5$^{KI/+}$* (n=5) and *Tie2-Cre;ADAMTS5$^{KI/+}$*(n=4) whole embryos at E9.5. Data are presented as mean ± SEM, and statistical significance was determined using an unpaired Student's *t*-test. **P<0.01. **C)** Co-immunostaining **of** CD31 and GFP on sections of E9.5 *ADAMTS5$^{KI/+}$* and *Tie2-Cre;ADAMTS5$^{KI/+}$* embryos. Higher magnification views are shown in the right panels. The white arrows indicate dorsal aorta. Scale bars: 200µm. **D)** Representative stereomicroscopic images of *Tie2-Cre;ADAMTS5$^{KI/+}$* embryos and littermate controls with yolk sac at E9.5. Scale bars: 1mm.

Disease Models & Mechanisms • Supplementary information

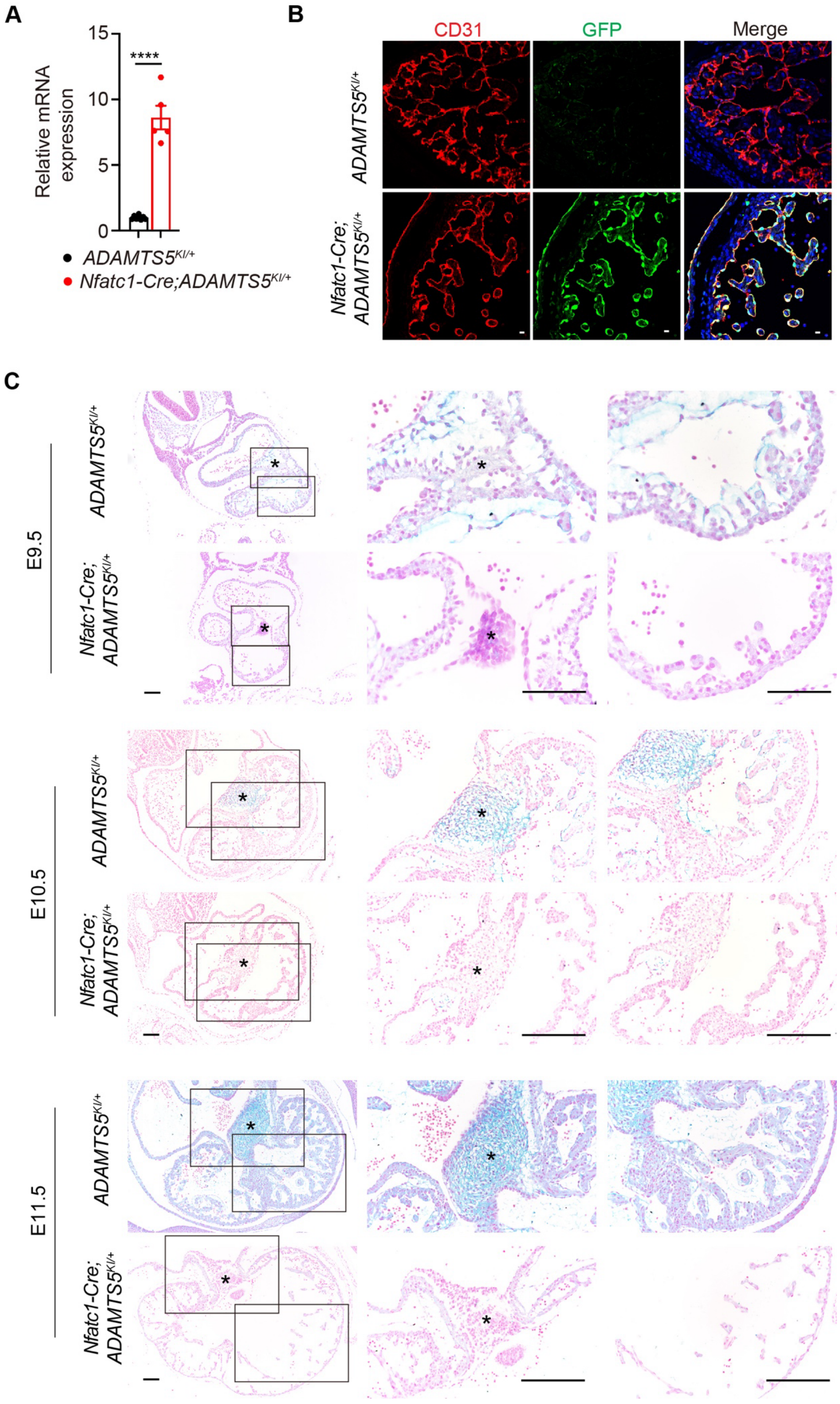
A
15
****
10
5
0
Relative mRNA expression
ADAMTS5KI/+
Nfatc1-Cre;ADAMTS5KI/+
B
CD31
GFP
Merge
ADAMTS5KI/+
Nfatc1-Cre; ADAMTS5KI/+
C
E9.5
E10.5
E11.5
ADAMTS5KI/+
Nfatc1-Cre; ADAMTS5KI/+

Fig. S2. Efficient ADAMTS5 expression and reduced proteoglycan levels during heart development in *Nfatc1-Cre; ADAMTS5*$^{KI/+}$ mice. A) Relative ADAMTS5 mRNA expression level of E11.5 *ADAMTS5*$^{KI/+}$ (n=8) and *Nfact1-Cre;ADAMTS5*$^{KI/+}$ (n=5) heart. Data are presented as mean ± SEM, and statistical significance was determined using an unpaired Student's *t*-test. ****P<0.0001. **B)** Co-immunostaining of CD31 and GFP on sections of E11.5 *ADAMTS5*$^{KI/+}$ and *Nfatc1-Cre;ADAMTS5*$^{KI/+}$ hearts. Scale bars: 10μm. **C)** Alcian blue staining of embryos at E9.5, E10.5, and E11.5 showing reduced cardiac jelly at AV cushion and trabeculae myocardium of *Nfact1-Cre;ADAMTS5*$^{KI/+}$ hearts as presented for main Fig. 2A-C. The right panel shows the higher magnification images. Representative images from at least 3 or more independent experiments are shown. The asterisk indicates the AV cushion. Scale bars:100μm.

Disease Models & Mechanisms • Supplementary information

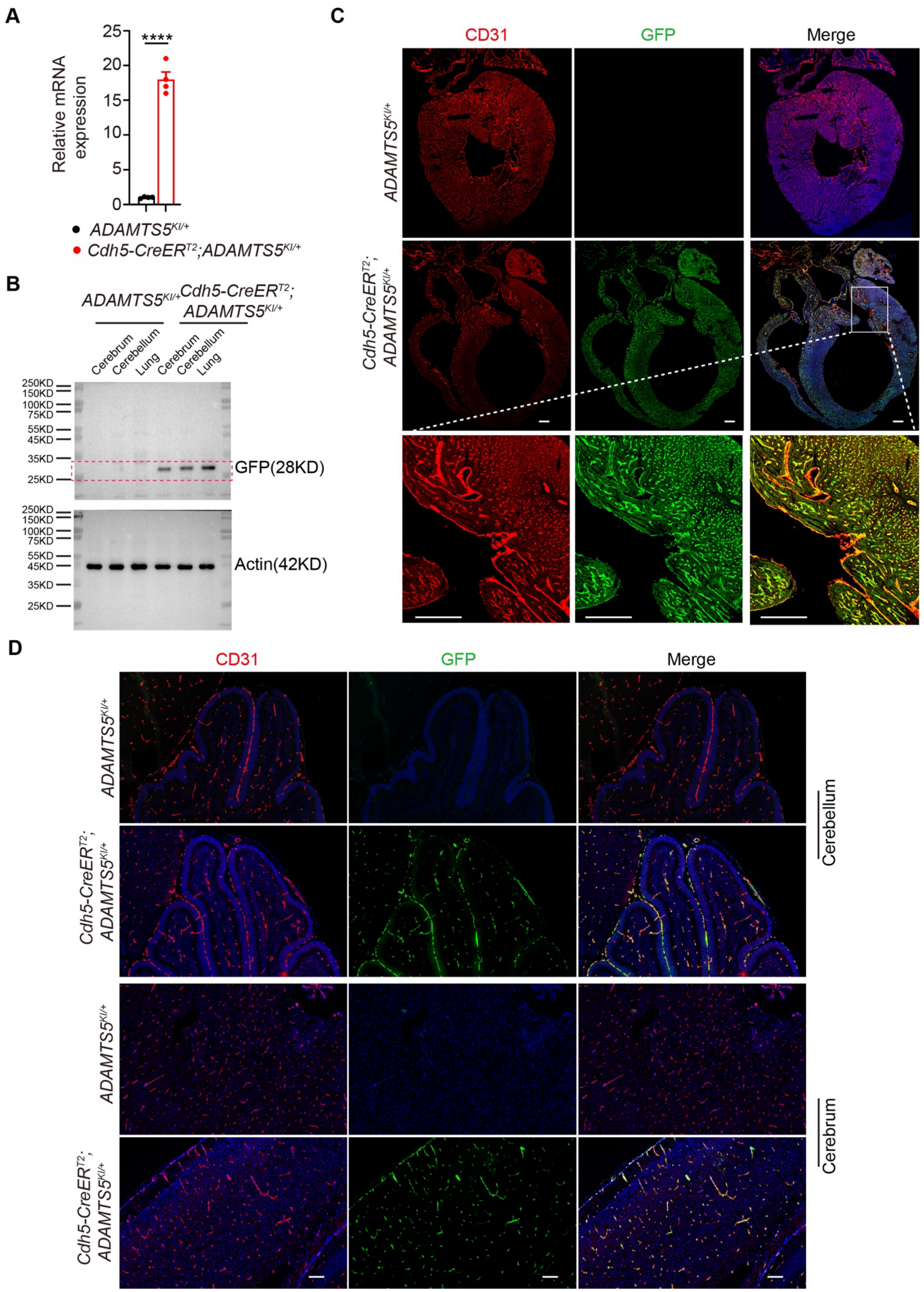

Disease Models & Mechanisms • Supplementary information

Fig. S3. Induction of ADAMTS5 expression in *Cdh5-CreER^{T2};ADAMTS5$^{KI/+}$* mice following 4-hydroxytamoxifen (4-HT) treatment. A) Relative mRNA expression levels of *ADAMTS5* in lung tissue from 4HT-treated *Cdh5-CreER^{T2};ADAMTS5$^{KI/+}$* (n=4) and *ADAMTS5$^{KI/+}$*(n=4) mice. Data are presented as mean ± SEM, and statistical significance was determined using an unpaired Student's *t*-test. ****P<0.0001. **B)** Full-length Western Immunoblots showing GFP protein level in lysates of cerebrum, cerebellum, and lung from 4-HT-treated *Cdh5-CreER^{T2};ADAMTS5$^{KI/+}$* mice. **C)** Co-immunostaining CD31 and GFP on heart sections of 4-HT-treated *ADAMTS5$^{KI/+}$* and *Cdh5-CreER^{T2};ADAMTS5$^{KI/+}$* mice. The bottom panels show the higher magnification views. Scale bars: 200μm. **D)** Co-immunostaining CD31 and GFP of brain sections of 4-HT-treated *ADAMTS5$^{KI/+}$* and *Cdh5-CreER^{T2};ADAMTS5$^{KI/+}$* mice. Scale bars: 100μm.

Disease Models & Mechanisms • Supplementary information

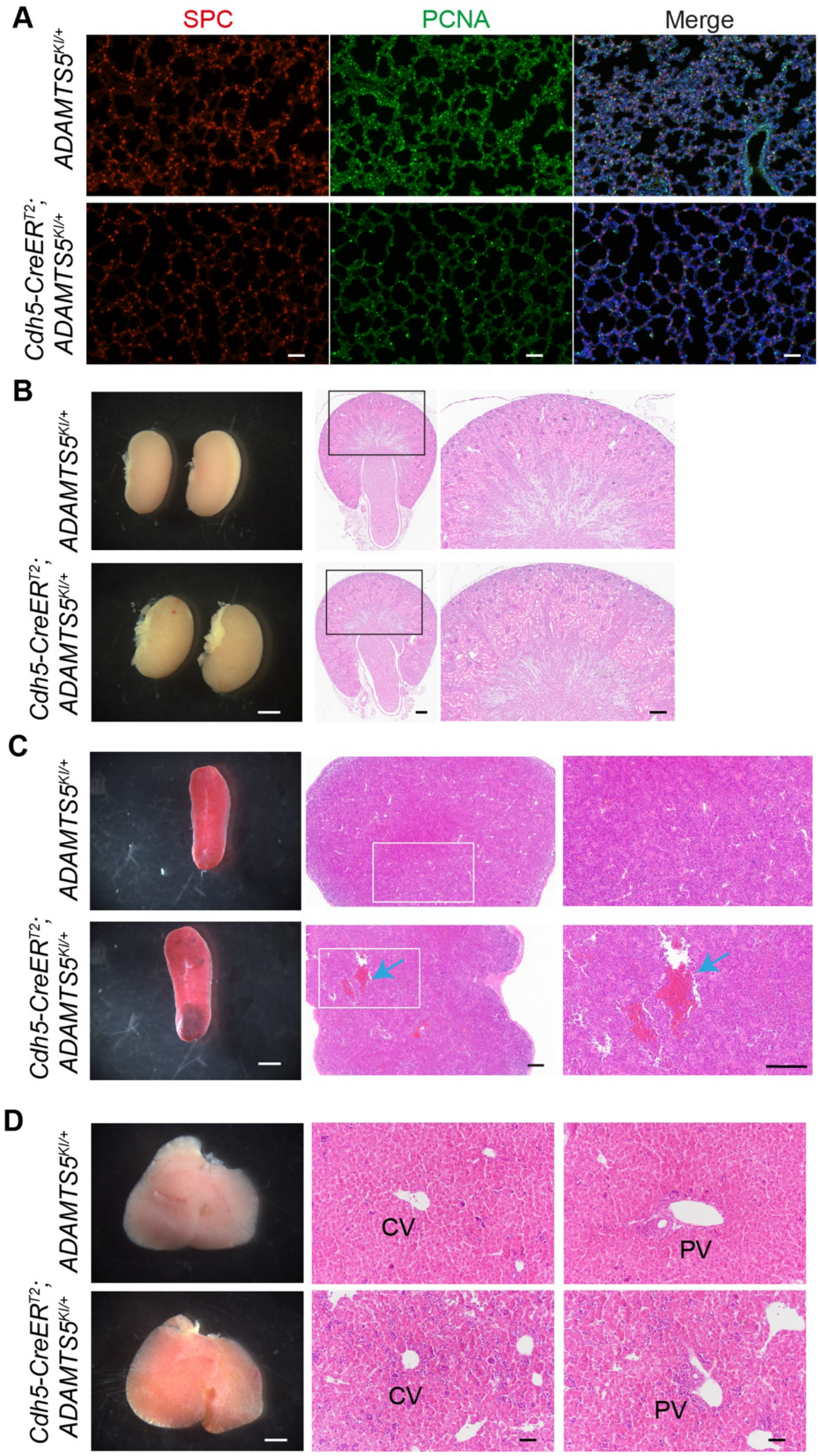

Fig. S4. Immunostaining and H&E staining of organ sections from mice with induced expression of ADAMTS5 in the endothelial lineage. A) Immunostaining of lung sections shows reduced SPC and PCNA expressing cells in the lung of induced *Cdh5-CreER*^{T2}*;ADAMTS5*$^{KI/+}$ mice

Disease Models & Mechanisms • Supplementary information

compared with littermate controls. **B-D)** Representative micrograph and H&E staining of kidney (**B**), spleen (**C**), and liver (**D**) from induced *Cdh5-CreER*^{T2}*;ADAMTS5*$^{KI/+}$ and *ADAMTS5*$^{KI/+}$ mice at P8. H&E staining detected increased bleeding in the spleen (blue arrow, **C**) and increased leukocyte infiltration in the liver (**D**) of induced *Cdh5-CreER*^{T2}*;ADAMTS5*$^{KI/+}$ mice. CV, central vein; and PV, portal vein. Micrographs scale bars: 2mm. H&E and immunostaining image scale bars: 100µm.

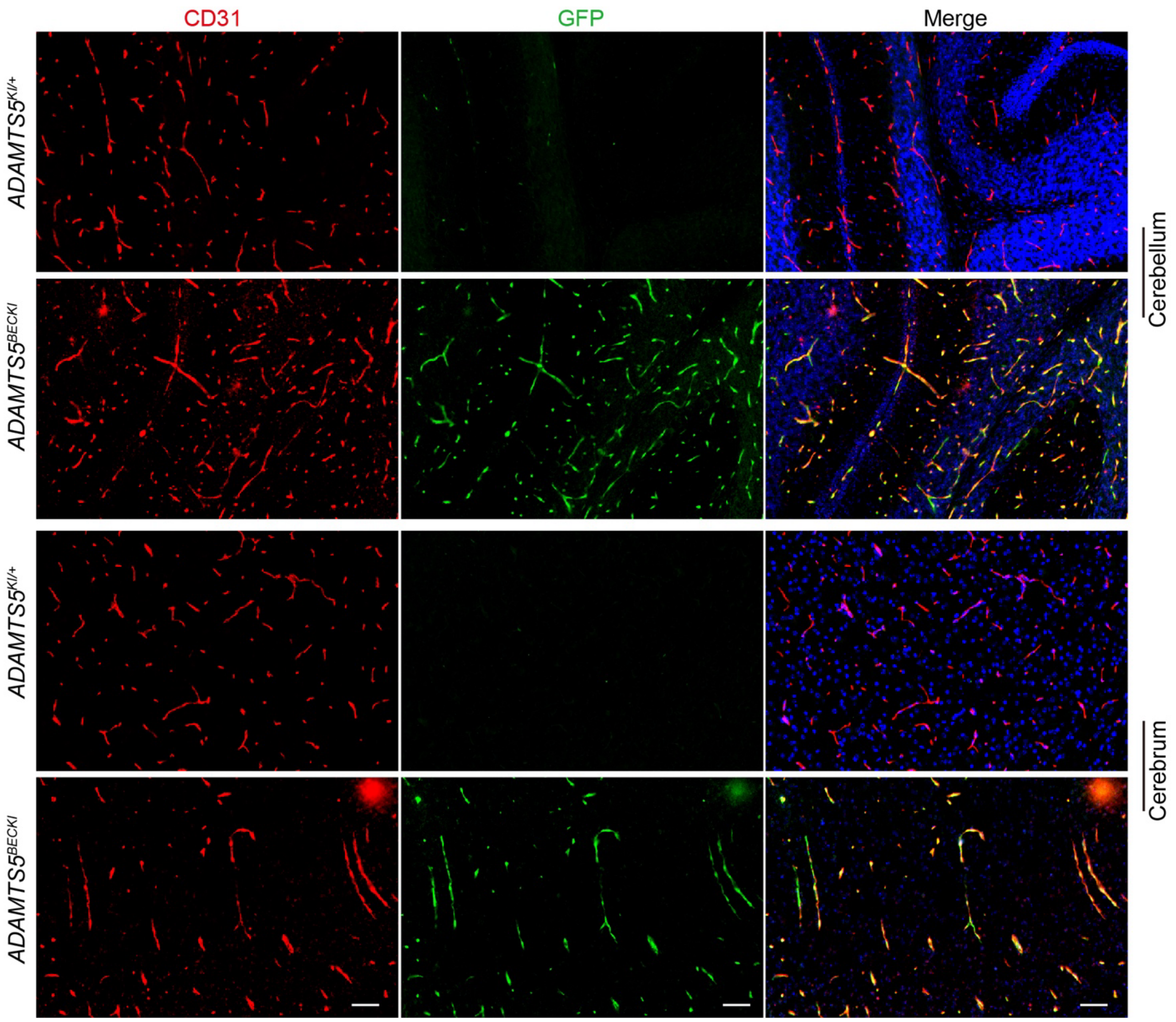

Fig. S5. The ADAMTS5 expression efficiency in *ADAMTS5^{BECKI}* mice. Co-immunostaining of CD31 and GFP on the brain section of *ADAMTS5^{BECKI}* and littermate control mice. Scale bars: 100µm.

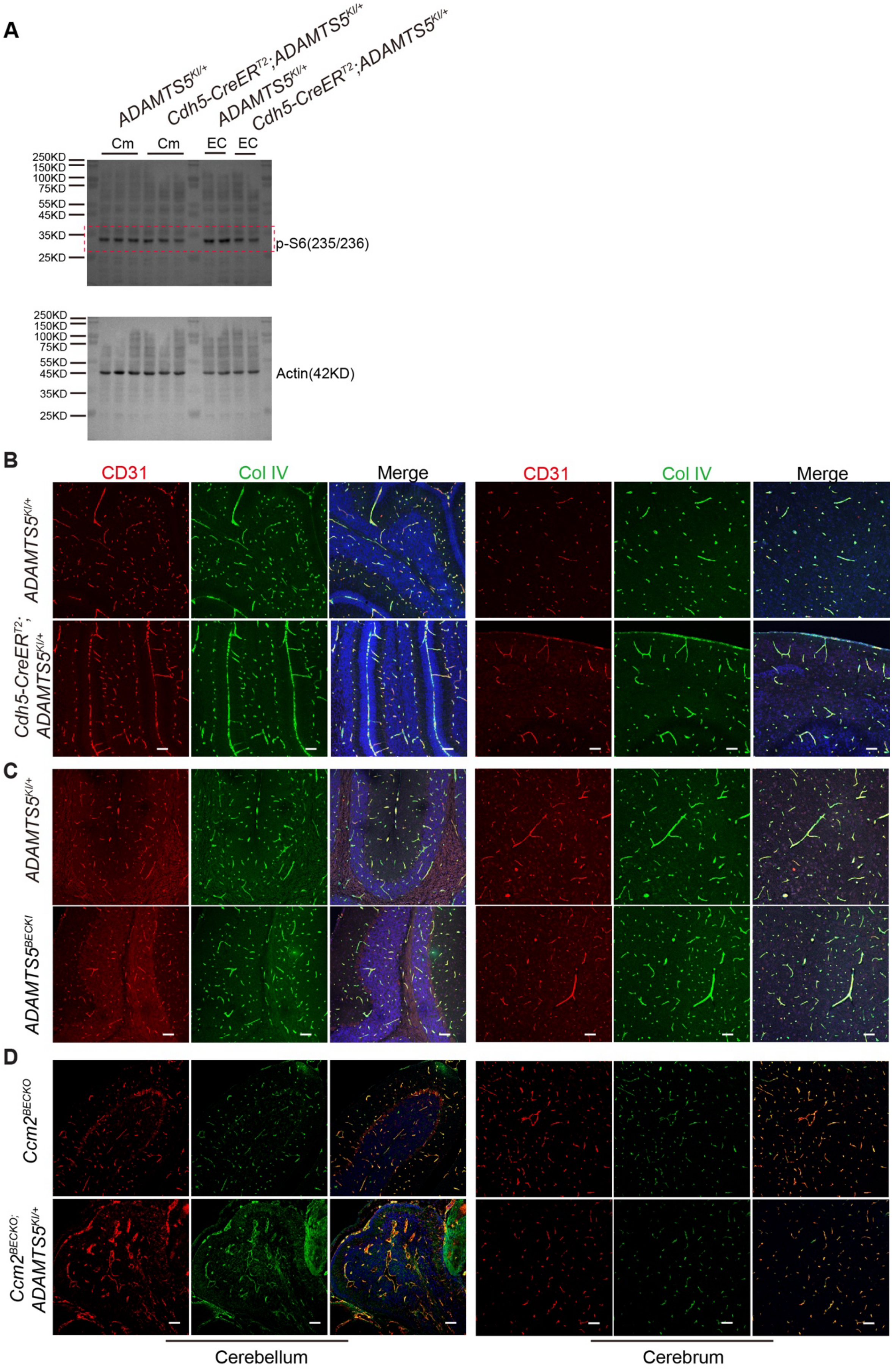
A
ADAMTS5KI/+
Cdh5-CreERT2;ADAMTS5KI/+
ADAMTS5KI/+
Cdh5-CreERT2;ADAMTS5KI/+
Cm
Cm
EC
EC
250KD
150KD
100KD
75KD
55KD
45KD
35KD
25KD
p-S6(235/236)
Actin(42KD)
B
CD31
Col IV
Merge
ADAMTS5KI/+
Cdh5-CreERT2;
ADAMTS5KI/+
C
ADAMTS5KI/+
ADAMTS5BECKI
D
Ccm2BECKO
Ccm2BECKO;
ADAMTS5KI/+
Cerebellum
Cerebrum

Fig. S6. p-S6 and Col IV expression in *ADAMTS5* knockin mice. A) Western blots showing p-S6 levels in lysates from cerebellum tissue and isolated cerebellar endothelial cells of 4-hydroxytamoxifen-treated *ADAMTS5*$^{KI/+}$ and *Cdh5-CreER*^{T2}*;ADAMTS5*$^{KI/+}$ mice. **B-D)** Co-immnunostaining of Col IV and CD31 assessing vessel integrity in *Cdh5CreERT2;ADAMTS5*$^{KI/+}$ **(B)**, *ADAMTS5*^{BECKI} **(C)**, and *Ccm2*^{BECKO}*;ADAMTS5*$^{KI/+}$ **(D)** mice and their littermate controls. Scale bars: 50μm.
